# Dual‐Function Lipid‐Based Nanovector Strategy for Glioblastoma Immunotherapy: STING Activation and M1 Microglia Polarization

**DOI:** 10.1002/ddr.70312

**Published:** 2026-05-12

**Authors:** Mohamed S. Nafie, Mohamed Khaled Diab, Sherif Ashraf Fahmy

**Affiliations:** ^1^ Department of Chemistry, College of Sciences University of Sharjah Sharjah United Arab Emirates; ^2^ Bioinformatics and Functional Genomics Research Group, Research Institute of Sciences and Engineering (RISE) University of Sharjah Sharjah United Arab Emirates; ^3^ Chemistry Department, Faculty of Science Suez Canal University Ismailia Egypt; ^4^ Pest Physiology Department Plant Protection Research Institute, Agricultural Research Center Giza Egypt; ^5^ Department of Pharmacy, Institute of Pharmaceutics and Biopharmaceutics Philipps University of Marburg Marburg Germany

**Keywords:** blood–brain barrier (BBB) penetration, cGAS‐STING signaling, cyclic dinucleotide STING agonists, glioblastoma immunotherapy, lipid‐based nanovectors, microglia reprogramming, thermo‐magnetic drug delivery, tumor microenvironment modulation

## Abstract

Glioblastoma (GBM) remains one of the most lethal brain malignancies because of its highly immunosuppressive tumor microenvironment and the limited penetration of therapeutics across the blood‐brain barrier (BBB). Although recent studies have separately explored STING agonism, microglial reprogramming, and nanocarrier‐based drug delivery, an integrated framework combining these strategies for GBM immunotherapy is still lacking. In this review, we present a new dual‐function lipid‐based nanovector (LNV) strategy that simultaneously activates the cGAS–STING pathway and induces tumor‐associated microglia to repolarize toward the antitumor M1 phenotype. In contrast to previous reviews, which address them as individual approaches, herein we consolidate these into an integrated therapeutic paradigm and provide a rational design roadmap based on drug cargo selection, lipid isoform composition, BBB targeting, and thermo‐/magnetically responsive release. We summarize how STING activation enhances type I interferon signaling, dendritic cell maturation, and cytotoxic T‐cell priming, while M1‐polarized microglia potentiate local inflammatory and phagocytic antitumor responses. In addition, we summarize the current nanocarrier platforms, preclinical evidence, and translational design considerations pertinent to this combinatorial approach. This review provides a conceptually integrated overview of the potential of dual‐action lipid nanovectors to overcome clinically relevant immunological and delivery barriers in GBM, as well as future directions for next‐generation nano‐immunotherapies.

**Abbreviations**
A3AR: adenosine A3 receptor
BBB: blood–brain barrier
cGAMP: cyclic guanosine monophosphate‐adenosine monophosphate
cGAS: cyclic guanosine monophosphate‐adenosine monophosphate synthase
CNS: central nervous system
CSCs: cancer stem cells
CTL: cytotoxic T lymphocyte
CTLA‐4: cytotoxic T‐lymphocyte‐associated protein 4
CTLs: cytotoxic T lymphocytes
c‐di‐AMP: cyclic dinucleotide cyclic diadenosine monophosphate
DCs: dendritic cells
GBM: glioblastoma
HRR: homologous recombination repair
Hsp27: heat shock protein 27
ICIs: immune checkpoint inhibitors
IFN: type I interferon
IFN‐γ: intratumoral interferon‐γ
IRF3: interferon regulatory factor 3
LNV: lipid‐based nanovector
MAPK: mitogen‐activated protein kinase
NF‐κB: nuclear factor kappa B
NK: natural killer
OVA: immunogenic tumor antigen ovalbumin
PD‐L1: programmed cell death ligand 1
RIG‐I: retinoic acid‐inducible gene I
RNAi: RNA interference
SCC: squamous cell carcinoma
siRNA: small interfering RNA
STING: stimulator of interferon genes
TAA: tumor‐associated antigens
TAM: tumor‐associated macrophage
TLR: toll‐like receptor

## 1 Introduction

Glioblastoma multiforme (GBM) is one of the most aggressive cancers. It is also the most common primary malignant brain tumor in young adults (Schaff and Mellinghoff 2023). Despite improving overall survival rates for many cancers, the advances have not yet translated to glioblastoma. Patients rarely survive beyond 15 months after diagnosis (Verdugo et al. 2022). The significant barriers for developing glioblastoma immunotherapy are the presence of an immunosuppressive glioblastoma microenvironment (GBM‐M) and the difficulty in delivering therapeutic agents across the blood–brain barrier (BBB) to target glioblastoma lesions (Wang, Liu, et al. 2022). The dense tumor mass covered by an intact BBB restricts the penetration of therapeutics and engineered immune cells. The systemically injected drugs quickly degrade without proper anchoring to the tumor site. Paradoxically, actively delivered agents can even accelerate tumor growth and grant cells resistance to treatments (Wu et al. 2023).

The primary immune barriers operating in GBM‐M involve the recruitment and accumulation of immunosuppressive regulatory T cells (Tregs) and pro‐tumor M2 microglia. They severely impair the efficacy of currently developed immuno‐oncology strategies (Zhao et al. 2025).

**Figure 1:**
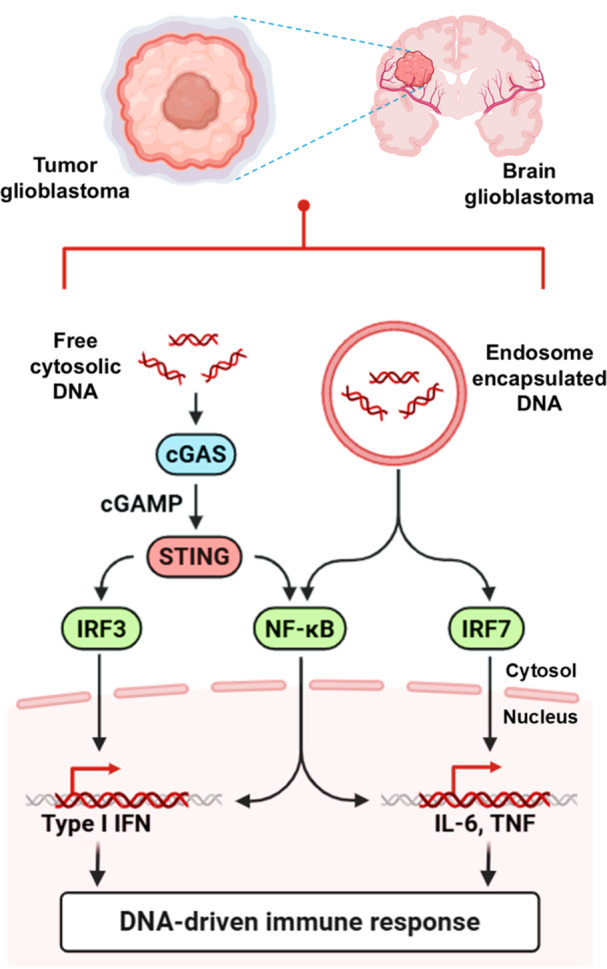
cGAS–cGAMP–STING pathway in GBM. Created in BioRender. https://BioRender.com/. The diagram illustrates the DNA‐driven immune response in glioblastoma. The figure shows a brain tumor in which free cytosolic DNA and endosome‐encapsulated DNA activated pathways. Free DNA activates cGAS, which generates cGAMP that then activates STING. This route induces the activation of IRF3, NF‐kB, and IRF7 to lead to Type I IFN generation, as well as IL‐6 and TNF, which trigger an immune response.

Immunotherapeutic strategies for GBM, including immune checkpoint blockade, vaccine‐based approaches, and oncolytic virotherapy, have shown encouraging activity in selected preclinical settings; however, their overall efficacy remains limited in patients because of the profoundly immunosuppressive tumor microenvironment and the restricted delivery of therapeutic agents across the BBB (Yang 2025). In particular, the enrichment of regulatory T cells, myeloid‐derived suppressor cells, and M2‐like microglia/macrophages suppresses effective antitumor immunity and reduces GBM responsiveness to currently available immunotherapies (Salvato and Marchini 2024). These limitations have prompted growing interest in therapeutic strategies capable of both restoring innate immune activation and remodeling the local tumor microenvironment. Among these, activation of the cGAS‐STING pathway has emerged as a promising approach because it promotes type I interferon production, dendritic cell maturation, and cytotoxic T‐cell priming (Khorramdelazad et al. 2026). At the same time, reprogramming tumor‐associated microglia from a pro‐tumoral M2‐like phenotype toward an inflammatory M1‐like phenotype may further reinforce local antitumor immunity and counteract immune suppression within the brain tumor niche (Fu et al. 2025). Nanovectors made from lipids represent an appealing platform for capturing these complementary features because they can be designed to encapsulate diverse immunomodulatory cargos, stabilize fragile molecules, and enhance tumor targeting (Zhang, Qu, et al. 2025). These BBB‐targeting strategies and stimuli‐responsive release systems can be combined with the aforementioned platforms to provide a rational approach to the spatial and temporal co‐delivery of STING agonists and microglia‐modulating agents (Ying et al. 2024; Gong et al. 2026), as shown in Figure 1. Accordingly, this review presents the novel idea of a double‐action lipidic nanovector for the delivery of GBM immunotherapy via STING pathway activation and M1 microglial polarization (Wang, Wang, et al. 2025; Zhang, Ji, et al 2025). We first highlight the key immunological and delivery impediments in GBM, then describe how lipid nanocarriers, thermo‐magnetic, and targeted delivery regimens can aid drug penetration into tumor tissue, followed by a critical assessment of the roles of the cGAS‐STING axis and microglial polarization in the GBM‐M. Finally, we summarize the design principles, cargo‐selection strategies, and translational challenges associated with engineering dual‐function nanovector systems for next‐generation GBM immunotherapy.

## 2 Glioblastoma and Immunotherapy: Barriers and Opportunities

Glioblastoma (GBM) is the most common malignant primary brain tumor, with an average survival of 15 months and less than a 10% 5‐year survival. GBM tumor development establishes an immunosuppressive milieu that limits immune recognition and promotes tumor antigen escape (Wang, Liu, et al. 2022). Accumulating aberrant tumor cells expressed the NKG2D ligand enigma, thereby attenuating the anti‐tumor immunity. Regulatory T cells (Treg) accumulated in the tumor mass and peripheral organs, restraining the activity of cytotoxic T lymphocytes (CTLs) and inhibiting the generation of effector T cells (Sheppard et al. 2018). Microglia exhibited a prominent M2‐like phenotype within the GBM. Upon sensing anti‐tumor immunity, GBM eventually enters an immune‐resistant state. This cascade transformation of an inflamed tumor to a resistant phase led to dramatic failure of checkpoint blockade therapy (Zhang, He, et al. 2025). The BBB impedes the entry of therapeutic agents into the tumor site. Furthermore, immunotherapy agents, including antibodies, cytokines, and immune checkpoint blockade, face similar delivery barriers and exhibit limited efficacy in GBM (Lechpammer et al. 2022).

GBM cells induce Treg influx, upregulate checkpoints, and polarize toward an M2 phenotype, which suppresses cytotoxic therapy (Qiu et al. 2023). The two‐faced lipid α‐aminophosphonate‐based nanovectors for GBM immuno‐oncology are summarized, including their effects on the STING pathway in combination with M1 microglial activity. The stimulator of interferon genes (STING) signaling pathway may also serve as a therapeutic target for GBM (Mu et al. 2024). STING agonists enhance the secretion of type I interferon (IFN) and pro‐inflammatory cytokines, including IL‐6, IL‐12, and CXCL10, which promote T cell and natural killer (NK) cell migration into the tumor (Sooreshjani et al. 2023). The STING signaling pathway induces the pro‐inflammatory immune phenotype in GBM. A growing body of evidence suggests that STING agonist‐treated GBM elicits robust antitumor immunity (Berger et al. 2022). The STING pathway enhances the maturation of dendritic cells (DCs) and prepares for tumoral neoantigen‐specific T cell activation. Polarization of microglia into the M1 phenotype showed strong anti‐tumor activity compared to the immunosuppressive (M2) phenotype (Tu et al. 2021). M1 microglia secreting several proinflammatory cytokines, such as TNF‐α, IL‐6, and IL‐1β, could effectively activate T‐cell immunity. Co‐delivering the STING agonist and signal 3 simultaneously, rather than delivering them separately, may enhance reliance on interferon (Battaglini et al. 2024).

Strategies for treating GBM, including antibodies, recombinant proteins, oncolytic viral therapy, and numerous small molecules, have been reported. The GBM microenvironment prevents T cell recognition and compromises the efficiency of immune targeting (Salvato and Marchini 2024). Different checkpoint molecules are upregulated, limiting the arsenal of anti‐checkpoint therapies. It is time‐sensitive to develop new combinations to note these forbidding strategies (Rodriguez et al. 2024). Scientists have investigated materials for surface modification and devices that can release drugs to circumvent the blood‐brain tumor barrier for drug delivery (Nozhat et al. 2023). Researchers are currently designing lipid‐based nanovectors specifically for GBM. Due to the unique physicochemical properties, such as simple preparation, high biocompatibility, extended drug loading capacity, and high drug encapsulation rate, lipid‐based nanovectors provide an alternative system for delivering cancer treatments for GBM (Gupta et al. 2023). Table 1 summarizes the key immune barriers that hinder the effectiveness of immunotherapy in GBM.

**Table 1:** Immunosuppressive signature pathways of the glioblastoma microenvironment combinatorial target therapy.

Feature	Description	Impact on immunotherapy	Targetable by the nanovector strategy	Relevant pathways/targets	Reference
Checkpoint molecule expression (PD‐L1, CTLA‐4)	GBM overexpresses checkpoint ligands to inhibit T cell activation	Leads to resistance to checkpoint blockade therapies	Through the co‐delivery of checkpoint blockade antibodies or inhibitors	PD‐1/PD‐L1, CTLA‐4	(Bao (2024))
Impaired T cell infiltration	Physical and molecular barriers prevent T cell entry into the tumor	Restricts immune surveillance and attack on the tumor	Through modulation of chemokines and IFNs	CXCL10, ICAM‐1, VCAM‐1	(Chen, Peng, et al. (2023))
Low antigen presentation	GBM cells poorly present tumor antigens to T cells	Reduces the effectiveness of T cell‐based therapies	Through enhancing dendritic cell activation through the STING pathway	MHC I/II, cGAS‐STING	(Low et al. (2024))
M2‐polarized microglia	Microglia adopt an M2 phenotype, promoting tumor progression and immune evasion.	Suppresses anti‐tumor immunity, promotes immune escape	Through STING agonists and M1‐polarizing agents	IL‐4, IL‐13, TGF‐β1, CD206	(Kuntzel and Bagnard (2022))
Myeloid‐derived suppressor cells (MDSCs)	MDSCs are recruited into GBM and suppress T cell responses	Contributes to an immunosuppressive milieu	Through cytokine modulation and STING activation	Arginase, IL‐6, IL‐10	(Beola et al. (2023))
Regulatory T cells (Tregs)	Tregs are recruited and expanded within GBM, dampening the effect of T‐cell activity	Suppressed CTL‐mediated tumor killing and compromised the efficacy of checkpoint inhibitors	Through the co‐delivery of siRNAs targeting Treg recruitment signals	TGF‐β, FoxP3, IL‐10	(Qiu et al. (2023))

## 3 Lipid‐Based Nanovectors in Oncology

**Figure 2:**
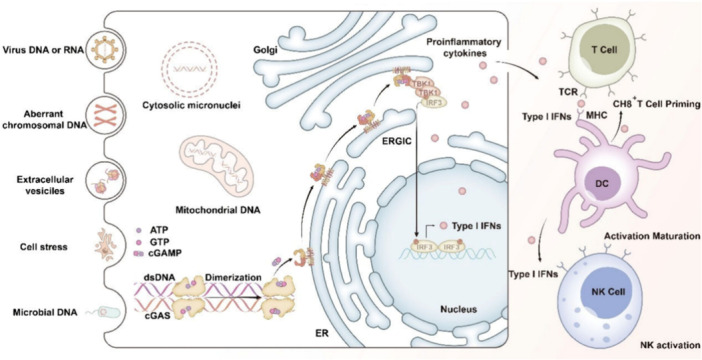
A diagram illustrates the cGAS‐STING signaling pathway for cancer immunotherapy (Guo et al. 2025). Copyright ACS Mol. Pharmaceutics 2025, 22, 12, 7262–7284. Schematic of the cGAS‐STING signaling pathway for cancer immunotherapy. It depicts the pathway initiated by different DNA materials, such as viral, faulty chromosomal, and microbial DNA, that leads to cGAS activation. This reaction, in turn, initiates cellular responses from the Golgi, ER, and ERGIC, thus leading to the production of proinflammatory cytokines and type I IFNs. The pathway leads to activation of T cells, dendritic cells (DCs), and natural killer (NK) cells.

Solid lipid nanoparticles for nucleic acid delivery in oncology might provide a new direction for cancer therapy. Nucleic acid signaling pathways are hijacked by cancer to evade immune detection and drive the growth, dissemination, and therapeutic resistance of cancer cells (Zhang, Rabinovsky, et al. 2025). Therapies that enhance nucleic acid detection and activate the cGAS‐STING pathway are being explored to improve immune surveillance across a wide variety of cancers, as schemed in Figure 2 (Guo et al. 2025). However, translating cGAS‐STING‐modulating therapies remains challenging (Huang et al. 2025). Initiating the cGAS‐STING pathway with STING agonists that are free of outdoor contaminants at ambient temperature may be efficient. However, these free cGAS‐STING pathway adjuvant molecules cannot selectively or persistently target unwanted biological compartments, leading to limited therapeutic benefits (Garland et al. 2022). Moreover, selected micro‐ and nano‐delivery carriers still cannot transport RNA and cGAS‐STING pathway‐modulating small molecules with adequate efficacy and safety to cancers located within the central nervous system, such as glioblastoma. More robust delivery vectors are therefore needed to expand the use of these powerful therapeutic agents (Qiao et al. 2025).

Compared with PLA, PLGA, and other biodegradable polymers, solid lipids offer a unique low‐temperature, solvent‐free preparation route, hot‐melt microfluidics, to produce a safe, anhydrous carrier (Garg et al. 2022). Solid lipid nanoparticles that feature drug/mRNA and STING pathway signal activators, with adequate pharmacological efficiency and stability, have emerged as elegant strategies to modulate the cGAS‐STING pathway (Ying et al. 2024). Lipids of tail‐chain polymerization can produce solid lipid nanoparticles that efficiently co‐encapsulate adjuvants under ambient temperature. An increase in the cGAS‐STING pathway in glioblastomas was achieved using solid lipid nanoparticles with core lipid tail‐chain structures, which enable the encapsulation of macromolecular RNA and small‐molecule adjuvants (Jnaidi et al. 2020). Polyethylene glycolylation of the solid lipid nanoparticle surface was introduced to achieve an ultra‐long blood circulation half‐life and enhance the blood‐to‐tumor exposure ratio. Investigators have reported the construction of lipidic NPs that can repolarize intracranial glioblastoma cells into bona fide antigen‐presenting cells and subsequently activate the cGAS‐STING pathway (Mu et al. 2024; Shi et al. 2021). After dual lipid nano‐adjuvant administration, pre‐existing T‐cell immunity can elicit potent tumor control and immunity memory against glioblastoma. Nevertheless, systemic cybernetic activation of direct semi‐decomposable co‐encapsulation, transfection‐free pro‐cGAMP, and poly‐branch diamond‐stabbed DNA adjuvants coupled to strand displacement entails high delivery risks to other regions and consequently impedes central nervous system‐accessible tumor treatments (Bao 2024; Pucci et al. 2020; Fan 2023).

### 3.1 Principles of Lipid Nanocarriers

Due to their specific physicochemical properties, lipid‐based nanocarriers (e.g., liposomes, solid lipid nanoparticles, and nanostructured lipid carriers) have become a powerful tool for anticancer drug delivery, particularly in glioblastoma, the most common and aggressive brain tumor in adults (Jnaidi et al. 2020; Wafik Nabih et al. 2025). Size, surface charge, lipid composition, and rigidity govern their performance (Gupta et al. 2023; Fahmy et al. 2022). These vesicles can encapsulate hydrophilic or hydrophobic contents, such as siRNA, mRNA, proteins, and small‐molecule drugs; co‐encapsulate two different payloads; and prevent contents from leaking out (Viegas et al. 2023). Lipid formulations can be prepared using various methods, resulting in a wide range of designs (Gupta et al. 2025).

Lipid nanocarriers can encapsulate various cargos; smaller constructs can also be loaded with mRNA and siRNA, which are more challenging to incorporate into polymeric vectors, and they are functionalized with targeting ligands (Shetty et al. 2022). Combining drug classes on a single platform could generate complementary, additive, or synergistic effects, enabling multimodal cancer combination therapy (Yang 2020). A group described a lipid nanoparticle that can carry either a STING agonist or an additional immunomodulator, potentially delivering both intracellular activation of the STING pathway and immune coordination within a single formulation (Wang, Wang, et al. 2025).

Current formulations and methods of action, therefore, do not provide the level of coordination necessary to stimulate the desired immunostimulatory or tumor treatment regimens precisely (Salvato and Marchini 2024). A dual‐functional approach involving the co‐delivery of STING agonists and another immunomodulating agent by a single vector should yield high development and operational flexibility, allowing for precise spatiotemporal control and better crosstalk opportunities to open up other STING‐stimulant immune cells to communication between each other, or in close pairings that exert synergistic effects; alternatively, negative regulatory agents could also be delivered concomitantly into the treatment strategy as needed (Mu et al. 2024). This approach involves developing a single nanocarrier platform that delivers both classes of agents directly to the tumor microenvironment, enabling well‐coordinated, temporally controlled multimodal glioblastoma immunotherapy (Marei 2022). Table 2 offers a comparative view of nanocarrier designs used in GBM immunotherapy.

**Table 2:** Types of lipid‐based nanocarriers and their key features for GBM delivery.

Nanocarrier type	Core material	Cargo types	BBB penetration	Immunological targeting	Stimuli‐responsiveness	Reference
Chimeric exosomes	Natural lipid membranes	STING agonists, mRNA	Intrinsic BBB permeability	Personalized STING activation	Endogenous targeting	(Bao (2024))
Liposomes	Phospholipid bilayer	Small molecules, RNA, mRNA, and proteins	Enhanced via transferrin/angiopep‐2 ligands	STING activation and M1 polarization	Thermal, magnetic, and NIR light	(Beola et al. (2023))
Magnetic lipid nanoparticles	Lipid co‐stabilized with SPIONs (Superparamagnetic iron oxide nanoparticles)	STING agonists, Doxorubicin, siRNA	Magnetic targeting plus ligands	Thermal‐triggered STING and M1	Alternating magnetic field	(Pucci et al. (2020))
Nanostructured lipid carriers (NLCs)	Solid and liquid lipid mixture	siRNA, cGAMP, dual payloads	Functionalized lipids for brain delivery	Dual STING and M1 microglia	Phase transition and magnetic	(Wang, Liu, et al. (2022))
Solid lipid nanoparticles (SLNs)	Solid lipids (e.g., glyceryl behenate)	STING agonists, RNA, mRNA	PEGylation for extended circulation	STING activation and microglial engagement	Hot‐melt fluidics and ambient release	(Fan et al. (2023))

### 3.2 Thermo‐Magnetic Activation and Targeting

Superparamagnetic nanoparticles loaded into lipid carriers enable thermomagnetic activation, allowing targeted heat‐mediated release of therapeutic agents at precise times and locations to minimize off‐target effects. Superparamagnetic nanoparticles generate local heat when exposed to an alternating magnetic field, which, when coupled with an appropriate external stimulus, can be used to control a release profile (Hegde et al. 2022). Thermo‐magnetic activation can be used with a variety of agents, enabling the simultaneous delivery of multiple therapeutic modalities. This approach avoids the problems that arise from using only cargo‐formulation strategies and provides greater control to improve the simultaneous delivery of multiple agents (Wang, Sun, et al. 2025).

**Figure 3:**
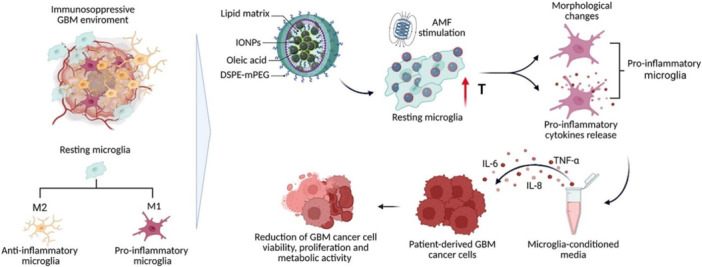
Lipid nanovectors for thermo‐magnetic stimulation in microglia cells in the context of glioblastoma therapy (Ceccarelli et al. 2025). Copyright ACS Appl. Mater. Interfaces 2025, 17, 46, 63253‐63271. Graphical representation of microglial activation and its consequences for GBM cancer cells. The model, based on microglia, is initiated by a resting microglial cell in an immunosuppressive GBM environment. Resting microglia become pro‐inflammatory microglia by the activation of AMF, which releases cytokines after AMF stimulation. The chart also shows the differentiation of resting microglia into anti‐inflammatory (M2) and pro‐inflammatory (M1) microglia. Key components include lipid matrix, IONPs, oleic acid, and DSPE‐mPEG. Cytokines IL‐6, IL‐8, and TNF‐α are released into microglia‐conditioned media.

The thermomagnetic delivery approach has been successfully utilized with a wide range of therapeutic agents, including small molecules and nucleotides, as illustrated in Figure 3 (Ceccarelli et al. 2025). Hybrid fat–PEG nanocarriers containing super‐paramagnetic nanoparticles loaded with doxorubicin were developed to target glioblastoma using the transferrin receptor pathway and release the drug in response to an alternating magnetic field (Hegde et al. 2022). Co‐encapsulation of the heat shock protein 27 (Hsp27) small interfering RNA (siRNA) further exploited the exterior temperature increase to promote the release of sensitive macromolecules (Iglesia et al. 2019). The identification of Hsp27 as a target also highlighted the presence of an epithelial‐mesenchymal transition‐like phenotype during glioblastoma progression (Lampros et al. 2022). Liposomal vectors decorated with magnetic nanoparticles have also been developed for thermomagnetic delivery using alternating magnetic fields and near‐infrared light as dual triggers. The angiopep‐2 peptide is used to direct liposomal transport across the BBB via receptor‐mediated endocytosis, and it further promotes liposomal internalization via the low‐density lipoprotein receptor‐related protein 1 (Di Polidoro et al. 2022). Chlorin e6 can penetrate the BBB and glioblastoma cells, where it can then be released for PDT‐assisted near‐infrared light irradiation. The heat‐triggered increase of chlorin e6 release is even more pronounced in the presence of superparamagnetic nanoparticles (Pucci et al. 2025).

Recent work on glioblastoma‐specific delivery of the cyclic guanosine monophosphate‐adenosine monophosphate (cGAMP) stimulator of interferon genes (STING) agonist using transferrin‐affixed poly(ethylene glycol) block copolymer nanoparticles formulated from natural protein, engineered protein, and peptide nanoparticles is also cited as an example of a thermo‐magnetic cargo activation system for glioblastoma therapy (Fu et al. 2025; Qiao et al. 2025). These platforms use the heat generated from superparamagnetic iron oxide nanoparticles (SPIONs) to trigger dual cGAMP and dexamethasone release within glioblastoma (Li 2023). Upon magnetic stimulation, the nanoparticles facilitate cGAMP release from endosomes, thereby activating the STING pathway at the tumor site (Chen, Xu, et al. 2024). Furthermore, dexamethasone effectively suppresses potential inflammatory responses induced by STING activation. The striking mechanistic discrepancy associated with STING‐activation modalities additionally justifies the development of several independent approaches to enable the simple platform‐wise co‐delivery (Garland et al. 2022).

## 4 cGAS‐STING Pathway in Tumor Immunology

To mount an effective adaptive immune response, cytotoxic T cells must encounter the antigen and receive a co‐stimulatory signal, although they face several impediments within the GBM‐M (Nader et al. 2024). Tumors restrict CD8 + T cell infiltration, express proteasome subunits and co‐stimulatory ligands, but do not present classical tumor‐restricted neoantigens (Low et al. 2024). What is more, glioblastomas also restrict CD8^+^ T cell responses upon their arrival in the tumor bulk by reprogramming the antigen‐processing and presenting machinery and the network of co‐inhibitory signals. Tumor immune response modulation attempts to boost T cell infiltration, improve antigen presentation, and block the co‐inhibitory receptor signaling have failed to translate from mice to humans (Yu et al. 2023).

Microglia are the principal resident innate immune cells of the CNS and account for approximately 10% of brain mass. The interaction between microglia and the tumor in glioblastoma involves complex signaling pathways (Buonfiglioli and Hambardzumyan 2021). Transforming mutations and tumor‐secreted factors induce atypical properties and tissue‐specific microglial responses to the tumor. Recent evidence suggests that glioblastoma tumors harbor subtypes of microglia and that these cells exhibit striking genomic changes driven by the tumor (Zhao et al. 2025; Zhou et al. 2025). Depleting microglia in mouse models inhibits glioblastoma growth. Glioblastoma patients show increased abundance of an M2‐like microglial population and expression of M2‐like markers, which correlate with poor patient survival and enhance malignant behaviors in glioma cells (Zhang, He, et al. 2025). Consequently, transforming tumor‐associated microglia into a proinflammatory, anti‐glioma M1 state may offer an innovative approach to combat glioblastoma. It has been reported that exogenous IL‐12 skews the phenotype of glioma‐associated microglia toward an M1 phenotype. Microglial expression of the proinflammatory mediator NO promotes glioma cell death by altering the energy metabolism of glioma cells (Kuntzel and Bagnard 2022).

### 4.1 Mechanisms of STING‐Mediated Immunity

**Figure 4:**
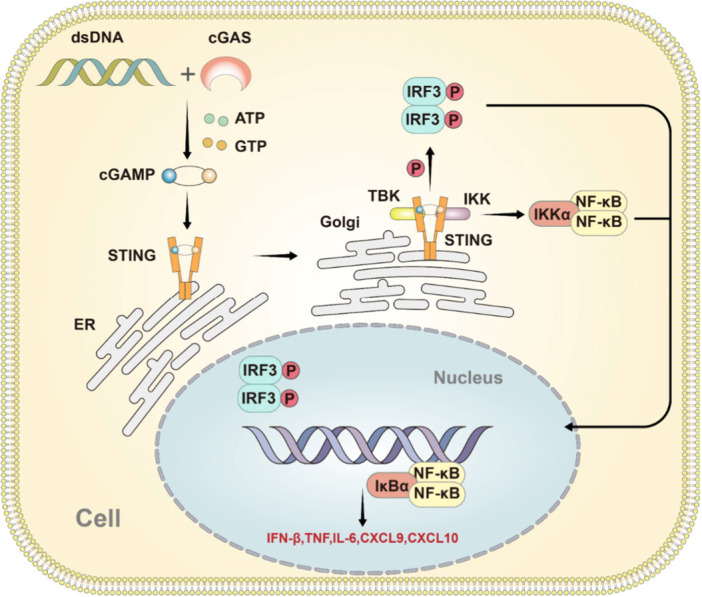
The cGAS‐STING pathway. Free cytoplasmic DNA, either of host or foreign origin, is sensed by cGAS to produce cGAMP with the use of ATP and GTP. cGAMP directly binds with STING at the ER to activate it, leading to shape mutation. The activated STING translocates to the Golgi, binds, and activates TBK1 and IKK via the IRF3 and NF‐κB pathways, thereby stimulating the expression of interferons (IFNs) and cytokines to enhance the immune response (Li, Xu, et al. 2025). Copyright ACS Omega 2025, 10, 12, 11723‐11742. Diagram illustrating the cGAS‐STING signaling pathway in a cell. Double‐stranded DNA (dsDNA) activates cGAS, which in turn produces cGAMP. This activates STING on the endoplasmic reticulum (ER), which then translocates to the Golgi. STING activation leads to the phosphorylation of IRF3 and the activation of IKK, which, in turn, activates NF‐κB. These signaling events result in the expression of genes such as IFN‐β, TNF, IL‐6, CXCL9, and CXCL10 in the nucleus.

With intratumoral delivery, a cGAMP‐encapsulated nanovector elicited a type I IFN‐dependent cytokine expression program in the tumor while also promoting DC maturation and the infiltration, expansion, and activation of antigen‐specific CD8^+^ T cells (Chen, Peng, et al. 2023). Previous studies demonstrated that glioblastoma‐released factors suppressed the expression of cGAMP‐responsive genes in immune cells (Low et al. 2024). Subsequent work demonstrated that glioblastoma cancer‐associated fibroblasts suppressed the expression of cGAMP‐induced genes, including Irf7 and Cxcl10, thereby blocking T cell priming. These findings underscore the importance of targeting the tumor microenvironment when developing effective STING‐based therapeutics for glioblastoma, as shown in Figure 4 (Li, Xu, et al. 2025).

### 4.2 Evidence for STING Activation in Glioblastoma

**Figure 5:**
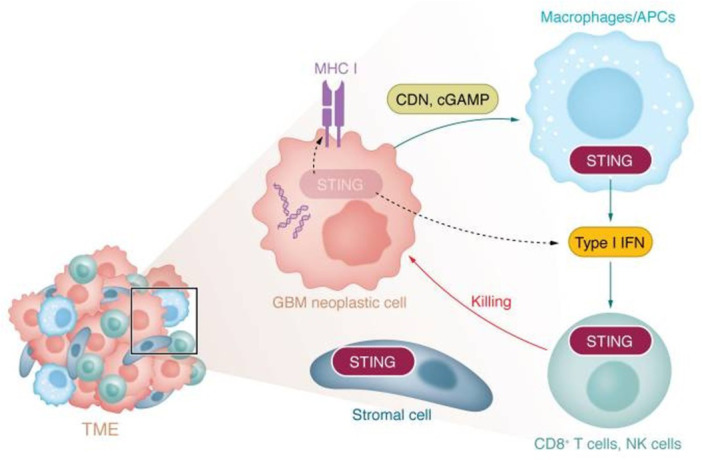
A framework for compartment‐specific involvement of STING signaling in the glioblastoma tumor microenvironment (Low et al. 2024). Copyright J Clin Invest. 2024, 134, 2, e163452. Schematic depiction of the STING signaling pathway in the glioblastoma microenvironment. It reveals the cross‐talk among GBM tumor cells, stromal cells, macrophages/APCs, and CD8^+^ T cells/NK cells. STING activation, MHC I, CDN, cGAMP, and Type I IFN are the most important elements constituting the procedure of cell killing.

The cyclic GMP–AMP synthase (cGAS)–STING pathway participates in cytosolic DNA sensing and immune responses against tumors. Signaling through the cGAS‐STING pathway leads to a type I interferon (IFN)–dependent program of gene expression that activates many chemokines, cytokines, and other immune genes (Islam et al. 2024). These immune mediators, such as those downstream of cGAS‐STING that are necessary for DC activation, and cGAS‐STING specifically, have become a focus of targeted pathways for glioblastoma treatment (Boudreau et al. 2021). Preclinical evidence demonstrated that in glioblastoma, different regimens and dosages of STING agonists led to enhanced immune responses and antitumor responses through activation of the cGAS‐STING pathway, as outlined in Figure 5 (Low et al. 2024).

**Figure 6:**
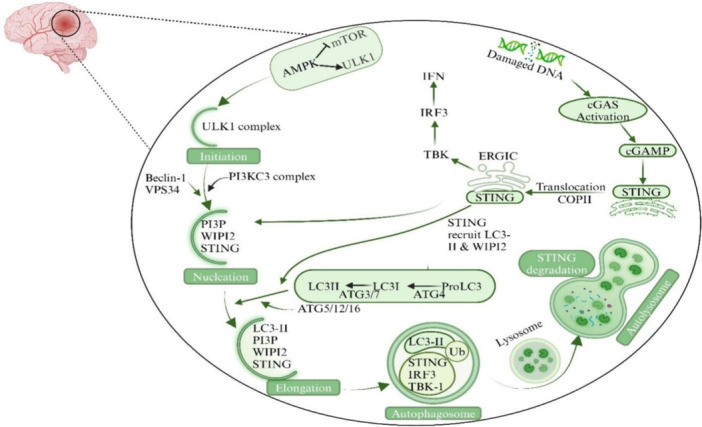
The crosslink between autophagy and the cGAS‐STING signaling pathway in TBI. The autophagy process is initiated by mTORC1 inhibition, which leads to ULK1 complex activation via AMPK signaling. The elongation of autophagic vesicles is mediated by PI3KC3, which acts in cooperation with the Beclin‐1/VPS34 complex. Another complex, ATG5/12, conjugates with ATG16L and integrates into autophagic vesicles. In addition, the endogenous dsDNA from mitochondria and nuclei recognized by cGAS also activated STING signaling, recruited LC3‐II and WIPI2, and was then engulfed into autophagosomes. The STING‐TBK1‐IRF3 complex may directly traffic to autophagosomes and fuse with lysosomes to form autolysosomes. cGAS‐STING activates autophagy, and this autophagic process promotes its degradation (Kumari et al. 2024). Copyright ACS Pharmacol. Transl. Sci. 2024, 7, 10, 2936–2950. This diagram illustrates the process of autophagy, which involves cellular self‐cannibalization in response to DNA damage. According to the flowchart, the process starts when DNA is damaged, leading to cGAS activation and cGAMP production. This step leads to the translocation and activation of STING, which, in turn, recruits proteins such as LC3‐II or WIPI2. The whole machinery consists of several complexes, such as ULK1 and PI3KC3, and is divided into initiation, nucleation, and elongation steps that form an autophagosome. The autophagosome finally fuses with a lysosome for degradation. They are annotated with key proteins and pathways that depict cell interactions and transitions. An inset shows a brain with an area highlighted, placing the process in its context.

Intratumoral administration of STING agonists is the most straightforward approach and has been studied in multiple preclinical glioblastoma models. These findings, together with the observations that the naturally occurring bacterial cyclic dinucleotide molecule c‐di‐AMP, its non‐nucleotide analogue 2ʹ3ʹ‐cyclic guanosine monophosphate‐adenosine monophosphate (GAMP), and the synthetic cyclic dinucleotide di‐GMP all trigger STING activation and immune responses within glioblastoma when administered intratumorally (Garland et al. 2022; Onyedibe et al. 2022). Nanoparticles encapsulating the STING agonist cGAMP administered via the intracranial route also induce cGAS‐STING activation and exert significant antitumor effects. After intratumoral delivery of STING agonists, the immune system is typically equipped to mount an effective immune attack against glioblastoma, as schemed in Figure 6 (Kumari et al. 2024).

Systemic supplementation with STING agonists has received attention for glioblastoma treatment due to practical drawbacks associated with the more direct methods (Mu et al. 2024). The systemic administration of the synthetic STING agonist 5‐3ʹ‐cyclic GMP‐AMP (3ʹ, 2ʹ‐cGAMP) alone is insufficient to activate the cGAS‐STING pathway (Berger et al. 2022). In contrast, combined administration with doxorubicin activates cGAS‐STING and elicits immune responses in immunocompetent glioblastoma models (Tankov et al. 2024). Intravenous administration of liposome‐loaded epitope‐specific vaccine (liposome‐MUC1 and liposome‐CAOV3) toward the extracellular domain of reciprocating murine T‐cell lymphoblastic leukemia virus surface glycoprotein in combination with a STING agonist was also demonstrated to be an effective treatment in syngeneic glioma models (Zhang 2023). However, the optimal agonist formulations that will confer satisfying and consistent therapeutic outcomes remain to be elucidated. Table 3 summarizes agonists, routes of delivery, and outcomes in GBM preclinical models.

**Table 3:** Comparison of STING agonists used in preclinical glioblastoma models.

Sr. No.	STING agonist	Type (CDN or small molecule/formulation)	Delivery method (in preclinical GBM model)	Immune effects (TME remodeling/innate immune activation/cell types)	Outcome in the GBM model	Combination therapy/limitations	Reference
1	ADU S100 (ML RR‐S2 CDA, MIW815)	Synthetic cyclic dinucleotide (CDN) (3′3′‐cGAMP analog)	Intratumoral injection in murine glioma models (e.g., GL261, CT‐2A)	Robust type I IFN response; increased innate immune cell infiltration (myeloid cells, likely microglia/macrophages), NK cell activation, pro‐inflammatory cytokines (e.g., IFN‐β, TNF‐α, IL‐6, CCL2), maturation of APCs, increased CD8^+^ T cell infiltration	Delay of tumor growth; improved survival compared to controls. In GL261/CT‐2A models, treatment‐induced TME remodeling and therapeutic benefit, in combination with radiotherapy or checkpoint blockade, show enhanced efficacy	Demonstrates proof‐of‐concept, but intracranial/intratumoral delivery may limit translational potential due to delivery challenges in human GBM	(Berger et al. (2022); Zaidi et al. (2021); Gan et al. (2022))
2	Antibody‐agonist conjugates/tumor‐targeted conjugates (e.g., STING agonist conjugated to tumor antigen targeting antibody)	Targeted conjugated STING agonist	Systemic (IV) or local, depending on design; targeted to tumor cells/TME via antibody binding	Localized STING activation in tumor milieu; increased APC activation, T cell infiltration, and immune memory; reduces off‐target systemic inflammation.	In preclinical solid‐tumor models, substantial antitumor effects, improved therapeutic window vs free STING agonists	Such a conjugate strategy could be adapted to GBM if conjugated to a glioma‐associated antigen (or tumor‐associated myeloid marker) and formulated in a nanovector/lipid system; aligning with dual function design (delivery + targeting + activation)	(Wu et al. (2022))
3	CDN‐loaded exosome formulations, e.g., exoSTING	CDN (or CDN analog) loaded into exosomes/vesicles	Intratumoral injection (as per current trials), possibly engineered exosomes for systemic delivery in the future	Enhanced STING activation in tumor‐resident immune cells; improved immune infiltration; reduced systemic exposure; better safety profile versus free CDN	Early‐phase clinical evaluation in advanced solid tumors; promising immunogenicity; may convert “cold” to “hot” tumors	Exosomes are particularly attractive for CNS delivery (due to natural ability to cross barriers), so exoSTING (or similar) may be highly relevant for GBM, especially with brain‐targeted or myeloid‐targeted exosomes	(Wang, Yu, et al. (2024))
4	Combination strategies: STING agonist + other therapies (e.g., radiotherapy, immunotherapy, checkpoint blockade)	Combinatorial therapeutic regimen/formulation	Varies; may use systemic or local delivery depending on components	Synergistic immune activation: STING‐mediated innate immunity plus radiation‐induced tumor cell death; more neoantigen release; enhanced T cell priming; improved TME remodeling; reversal of immunosuppressive myeloid cells; increased CD8^+^ T cell infiltration and function	In preclinical tumor models (non‐CNS), the combination often leads to more complete and durable tumor regression, immune memory, and sometimes better than monotherapy	For GBM, combining STING nanovector with standard‐of‐care (e.g., radiotherapy) or checkpoint blockade/adoptive therapy could maximize benefit, especially if the nanovector ensures delivery into the tumor/TME	(Shaik et al. (2025))
5	c‐di‐GMP (bacterial cyclic dinucleotide)	CDN	Intratumoral injection in glioma‐bearing mice (murine glioma)	Enhanced type I IFN signaling; increased T cell migration into brain/tumor; increased innate immune activation in TME	Significantly improved survival in glioma‐bearing mice compared with controls. Also, when combined with a peripheral vaccine, the survival benefit was greater than either alone	Demonstrates host STING (in immune compartment) is required: benefit lost in mice homozygous for nonfunctional “Golden ticket” (Gt) STING variant	(Low et al. (2024))
6	diABZI (non‐CDN small molecule STING agonist), delivered via specialized nanocarrier	Small‐molecule STING agonist, formulated within a bespoke lipid nanoparticle carrier (e.g., BLNP) targeting TAMC (tumor‐associated myeloid cells)	Intracranial (intratumoral) administration via implanted cannula into murine glioma (e.g., CT‐2A) brains; sometimes combined with radiotherapy (RT)	Potent TAMC (tumor‐associated myeloid cell) reprogramming: increased expression of co‐stimulatory molecules (CD40, CD80, CD86), enhanced antigen presentation, shift from immunosuppressive (Arg1^+^) to inflammatory (iNOS^+^) myeloid phenotype, increased production of T cell–recruiting/activating cytokines (e.g., CXCL10, CCL2), leading to strong CD8^+^ T cell infiltration and activation.	Dramatically enhanced antitumor efficacy when combined with radiotherapy: in the CT‐2A model, ~60% of mice became long‐term survivors (LTS), with no detectable tumor burden or brain toxicity. Also improved CAR T cell and adoptive CD8^+^ T cell infiltration into brain tumors, and long‐term immune memory (brain‐resident memory CD8^+^ T cells), and resistance to tumor rechallenge	Demonstrates that targeted delivery (lipid‐based nanocarrier) is key to overcoming the barriers of the BBB and achieving selective TAMC reprogramming, minimizing off‐target effects. Indicates a rational route for combining STING agonism with standard‐of‐care (RT) or adoptive cell therapies in GBM	(Zhang (2023))
7	Emerging nanovector‐based cGAS/STING modulating strategies (e.g., polymeric nanoparticles, liposomes, exosomes, metal ion co‐delivery, hybrid conjugates); general category rather than single agent	Delivery platform + STING agonist (or STING‐activating strategy)	Intratumoral, systemic, or BBB‐targeted, depending on design; flexible	Enhanced delivery, reduced degradation, improved uptake by immune cells (e.g., myeloid, microglia), better cytosolic delivery, improved retention, and local concentration	In preclinical non‐CNS tumor models, these strategies markedly improve the therapeutic index of STING agonists versus free (unformulated) agonists; increased efficacy, reduced toxicity, potential for systemic administration	This is essentially the targeted aim. Emphasizes that your “dual function lipid‐based nanovector” approach is not only conceptually sound but aligned with cutting‐edge developments	(Fu et al. (2025); Qiao et al. (2025))
8	Engineered bacteria‐based STING agonist vectors (e.g., bacteria engineered to produce CDNs in situ), e.g., SYNB1891 (vector)	Living bacterial vector producing STING agonist (e.g., cyclic di GMP) in the tumor microenvironment	Intratumoral injection/colonization of tumor tissue (preclinical/clinical solid tumors)	Sustained local production of CDN; persistent STING activation; type I IFN release; APC activation; enhanced CD8^+^ T cell infiltration; reprogramming of suppressive myeloid cells	Early‐phase clinical trials in advanced solid tumors, although not yet applied to CNS tumors, demonstrate the feasibility of in situ production of STING ligands.	For GBM, this is more speculative; brain safety, immune suppression, and blood‐brain barrier (BBB) issues might complicate use. However, conceptually interesting: combining bacterial tropism plus in situ STING activation and nanovector or targeting	(Wang, Yu, et al. (2024))
9	IACS‐8803 (novel/synthetic STING agonist)	Synthetic (small molecule or CDN‐like; per report)	In situ/intratumoral administration in preclinical glioblastoma models (including QPP8; possibly others)	Reprogramming of the glioma immune microenvironment (TIME), increased inflammatory innate immune responses, presumably involving myeloid cells/microglia/macrophages	Extended median survival in multiple preclinical GBM models, including immunocompetent and perhaps “cold” tumors; reportedly comparable or superior to ADU‐S100; enhanced anti‐tumor immunity	Encouraging for translation, but detailed immune cell–type analyses (e.g., M1 vs. M2 polarization, microglia vs. infiltrating macrophages) remain to be further characterized	(Najem et al. (2024); Nerdinger et al. (2025))
10	IACS‐8779	Next‐gen CDN similar to IACS 8803	Preclinical delivery, likely local/intratumoral in original studies	Potent STING activation; similar immunostimulatory profile as IACS‐8803 (CD8^+^ T cell activation, innate immune activation)	Tumor suppression/regression in preclinical models; robust systemic antitumor efficacy	Another CDN candidate that may outperform first‐gen CDN, with nanovector/lipid formulation and BBB‐permeable design, could be adapted to the GBM/CNS context	(Ager et al. (2019))
11	MK‐1454 (synthetic CDN analog)	CDN analog	Intratumoral (preclinical/clinical)	STING activation leads to type I IFN and immune activation in TME, APC maturation, and T cell infiltration (based on general CDN STING biology)	Preclinical tumor regression; entered early‐phase clinical trials (solid tumors/lymphomas); demonstrating feasibility in human cancer immunotherapy	As a clinically advanced CDN, MK‐1454 illustrates translational potential. But it is still likely to require local delivery, underscoring the need for improved delivery (e.g., nanocarrier) for CNS tumors.	(Gan et al. (2022); Wang, Meng (2024))
12	Nanoparticle/lipid‐based STING agonist delivery platforms (e.g., lipid nanoparticles, endosomolytic polymersomes, CDN‐loaded NPs)	Formulation/delivery strategy plus CDN (or small molecule) cargo	Intratumoral or potentially systemic, depending on design; nanoparticles improve cellular uptake, prolong retention, and enhance cytosolic delivery	Enhanced cytosolic delivery of CDN; increased STING activation; stronger type I IFN responses; improved APC activation/TME remodeling; better dendritic cell activation and T cell priming	In preclinical (non‐CNS) tumor models, demonstrated enhanced antitumor immunity versus free CDN; more potent, durable responses	This class/strategy is directly aligned with your proposed “dual function lipid‐based nanovector” concept. Potential to adapt such platforms for intracranial delivery or BBB targeting in GBM	(Qiao et al. (2025))
13	Non‐CDN small molecules: MSA‐2	Non‐CDN small‐molecule STING agonist	Systemically feasible (oral, subcutaneous, intratumoral) in preclinical models	STING pathway activation in TME; induction of pro‐inflammatory cytokines (IFN β, TNF‐α, IL‐6); reprogramming TME toward inflammation; M1‐like macrophage polarization in some models	Durable anti‐tumor immune responses in murine cancer models (non‐CNS) when administered systemically; more clinically practical than CDNs requiring intratumoral injection	Because of systemic delivery feasibility and small molecule nature, MSA‐2 (or similar) could be particularly attractive for GBM if a brain penetrant nanovector is designed (lipid‐based nanovector, perhaps with BBB‐targeting)	(Nerdinger et al. (2025))
14	STING‐agonistic cyclic‐dinucleotides (CDN) encapsulated in spherical nucleic acid (SNA) nanoparticles (e.g., 2′3’‐cGAMP or synthetic CDNs)	CDN (or CDN analog), “STING‐agonistic CDN.”	Intratumoral (intracranial) delivery of SNA nanoparticles in murine GBM models (preclinical)	Enhanced STING activation within the tumor (likely in myeloid cells/neighborhood), leading to innate immune activation, type I IFN induction, and remodeling of tumor microenvironment	Therapeutic benefit in GBM models: tumor regression/suppression; though authors note “inadequate” penetration/delivery remains a challenge in some settings	Shows potential of nanoparticle formulations to improve delivery of CDNs to intracranial tumors, but highlights remaining delivery/penetration barriers in GBM (e.g., diffusion, cell uptake, BBB)	(Mahajan et al. (2025))

## 5 Microglia Polarization in the Tumor Microenvironment

Microglial polarization in the GBM‐M is key to tumor immunology (Kuntzel and Bagnard 2022). Despite the association of glioblastoma with astrocytes, microglia are the most predominant immune cell type; they also maintain brain immunologic homeostasis and CNS development (Geribaldi‐Doldán et al. 2019; 2021). Like peripheral macrophages, microglia can undergo phenotypic and functional polarization. Two significant statuses are recognized: the M1 pro‐inflammatory status, which secretes TNF‐α, IL‐12, IL‐1β, and IL‐6, and the M2 anti‐inflammatory status, which promotes tumor growth and immune suppression. Central administration of IL‐4, IL‐10, and IL‐13 directs microglia toward the M2 phenotype (Zhang, He, et al. 2025; Ren et al. 2023).

**Figure 7:**
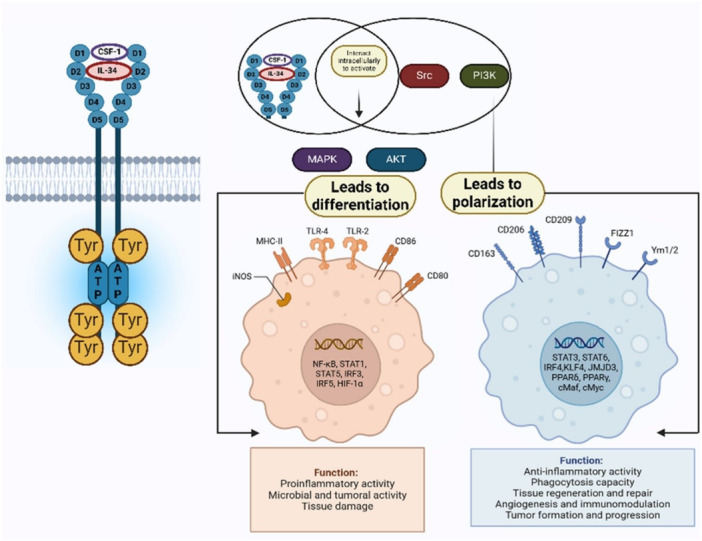
CSF‐1 controls M1 and M2 macrophage subtype differentiation and polarization (Naik et al. 2025). Copyright ACS Pharmacol. Transl. Sci. 2025, 8, 10, 3391‐3410. A flowchart showing the effects of CSF‐1 on macrophage lineage commitment and polarization. The chart depicts the signaling pathway triggered by CSF‐1 binding to its receptor, and the subsequent pathways involving Src, PI3K, MAPK, and AKT. This gives rise to M1 macrophages, which are pro‐inflammatory, and M2 macrophages, which are associated with anti‐inflammatory roles. The key proteins and receptors associated with each pathway are marked, with antigen‐related roles in immune response and tissue repair.

The effects of glioblastoma and microglia on each other are complex. The growth factors (GM‐CSF, IL‐34) secreted by tumor cells have the potential to drive an M2‐type that is supportive of tumors, as illustrated in Figure 7 (Naik et al. 2025). M2 microglia blocks local adaptive immunity by inducing the differentiation and expansion of FoxP3+ regulatory T cells, secreting immunosuppressive arginase, and inhibiting T‐cell cytotoxicity via PD‐L1. Glioblastoma also induces the microglia to produce pro‐inflammatory cytokines and chemokines. The balance of co‐operating polarization signals determines the overall microglial response in either a pro‐ or anti‐tumoral direction (Yi et al. 2025).

### 5.1 M1 Versus M2 Phenotypes and Immunomodulation

Polarized tumor‐associated macrophages (TAMs) are generated in the GBM‐M, predominantly the M2 phenotype, ultimately contributing to an immunosuppressive environment and promoting tumor growth (Ye et al. 2019). Several tactics have been published to drive M1 polarization of glioblastomas by targeting the relevant pathways or cytokines for monocyte recruitment and activation (e.g., TLR9, IL‐4, NF‐kB, and IL‐10), the pro‐inflammatory response (RACK1), or the inhibition of M2 marker expression (CD206; TGFb1, and TGFb2) (Wang, Liu, et al. 2022). However, these strategies have rarely been evaluated in combination with STING‐activating cGAMP delivery, suggesting that more systemic approaches should be considered when designing combinatorial strategies. Strategies leading to M1 polarization and STING activation are as yet undescribed in glioblastoma (Zhou et al. 2023).

### 5.2 Strategies to Induce M1 Polarization in Glioblastoma

To alter the immunosuppressive state in glioblastoma, several strategies have been tested to specifically and locally stimulate M1 polarization (Wang, Liu, et al. 2022). The Toll‐like receptor (TLR) pathway is one of the best‐known families for M1 reprogramming, which induces the M1 phenotype mainly through mitogen‐activated protein kinase (MAPK)/nuclear factor kappa B (NF‐κB) and interferon regulatory factor 3 (IRF3)‐mediated signaling pathways (Ye et al. 2019). With respect to the TLR ligands, those for TLR3 (e.g., polyinosinic–polycytidylic acid [poly(I: C)]), TLR7 (e.g., resiquimod), and TLR9 (CpG) have been reported to inhibit M2 tumors via anti‐M2 macrophage accumulation in glioblastomas (Bao 2024). It has been reported that an anti‐PD‐1 antibody, in combination with poly(I:C), IPI549, and CpG‐loaded nanoparticles, can stimulate the TLR pathway to induce M1 polarization (De Waele et al. 2021).

**Figure 8:**
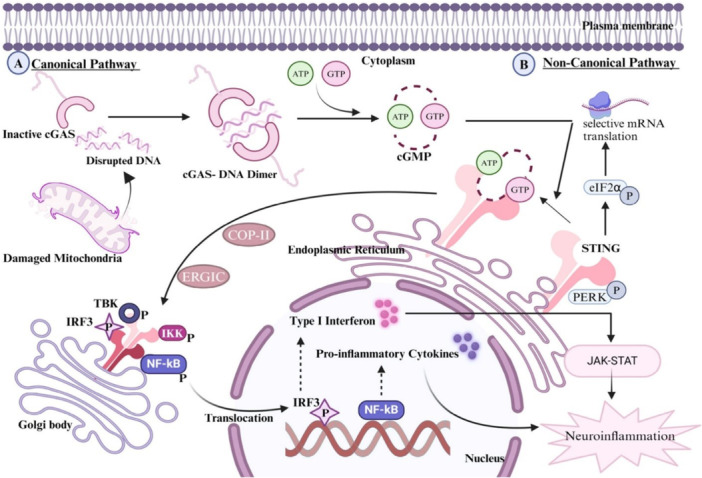
cGAS‐STING DNA sensing signaling pathway. (A) Canonical pathway: with the damaged DNA as a DAMP, cGAS recognizes it and forms a 2:2 complex. The binding events induce a conformational change in the active site of cGAS, leading to the production of 2′,3′‐cGAMP, a secondary messenger derived from ATP and GTP. 2′,3′‐cGAMP engages with the ER‐STING protein, which then relocates to the ER–Golgi intermediate compartment (ERGIC) and eventually to the Golgi apparatus. STING activates IKK and TBK1, which phosphorylate STING and subsequently recruit IRF3. The dimerization of IRF3 ultimately leads to its nuclear translocation and interaction with NF‐κB, thereby initiating type I IFN activation. Moreover, type I IFN promotes the activation of the JAK‐STAT pathway. The type I IFN, NF‐kβ, and JAK‐STAT pathways stimulate the secretion of pro‐inflammatory cytokines and chemokines, subsequently triggering the onset of neuroinflammation. (B) Noncanonical pathway: STING interacts with PERK via its intracellular domain, activating PERK. The PERK enzyme subsequently phosphorylates eIF2α, enhancing inflammatory translational activity. As a result, STING is separated from PERK and transported to ERGIC, where it is incorporated into the STING‐TBK1‐IRF3 complex (Kumari et al. 2024). Copyright ACS Pharmacol. Transl. Sci. 2024, 7, 10, 2936–2950. The cGAS‐STING DNA‐sensing signaling pathway is shown in the diagram. The figure is split into two parts: (A) Canonical pathway and (B) Non‐canonical pathway. In the classical pathway, damaged DNA that is delivered via cGAS forms a complex that generates 2’,3’‐cGAMP and binds to ER‐STING, thereby activating IKK and TBK1 kinases, recruiting IRF3 protein, and turning on NF‐kB, with production of type I IFN and pro‐inflammatory cytokines. In the non‐canonical route, STING associates with PERK, whose phosphorylation of eIF2α activates inflammatory translation and, subsequently, neuroinflammation. The pathways include several cellular organelles, such as the Golgi apparatus, endoplasmic reticulum, and nucleus.

**Figure 9:**
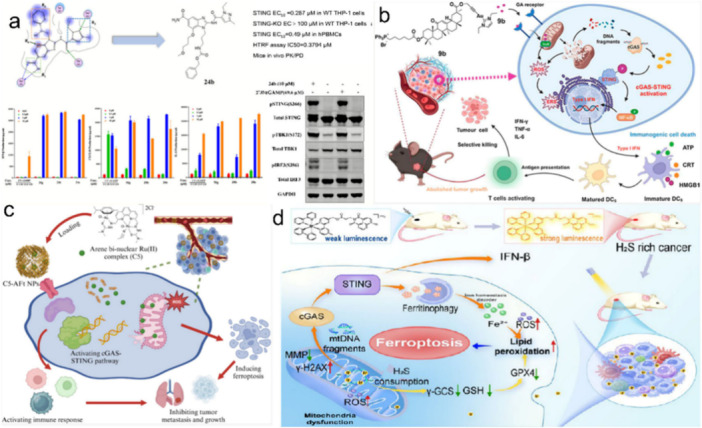
Diversified strategies for multifunctional chemical small molecules to activate the cGAS‐STING pathway: (a) Structural optimization facilitates direct STING activation; reproduced with permission from reference (Xi et al. 2020). Copyright ACS J. Med. Chem. 2020, 63, 1, 260–282. (b) A gold(I) complex triggers a mitochondrial surge of reactive oxygen species and causes DNA damage, thereby synergistically activating the cGAS‐STING pathway; Reproduced with permission from reference (Li, Wen, et al. 2024). Copyright ACS J. Med. Chem. 2024, 67, 3, 1982–2003. (c) An aromatic ruthenium (II) complex induces ferroptosis and DAMP release, thereby activating the cGAS‐STING pathway via dual mechanisms; Reproduced with permission from reference (Xu et al. 2024). Copyright ACS J. Med. Chem. 2024, 67, 21, 19573–19585. (d) Engineered molecules that respond to concentration enable spatiotemporal‐specific control; reproduced with permission from reference (Li, Liu, et al. 2024). Copyright ACS J. Med. Chem. 2024, 67, 18, 16235–16247.] (Guo et al. 2025). Copyright ACS Mol. Pharmaceutics 2025, 22, 12, 7262–7284. The flowchart illustrates diversified strategies for activating the cGAS‐STING pathway using small molecules. Panel (a) shows structural optimization of STING activation, including graphs and protein blots. Panel (b) represents a DNA lesion and reactive oxygen species induced by a gold(I) complex. Panel (c) presents an aromatic ruthenium complex‐driven ferroptosis and release of DAMP. Panel (d) highlights responsive molecular engineering for targeted regulation, involving diverse molecular interactions and cellular processes.

The activation of the cGAS‐STING pathway is another approach, which relies on two major signaling targets, cyclic GMP‐AMP synthase (cGAS) and STING, to enhance the M1 phenotype and trigger a systemic adaptive immune response (Samson and Ablasser 2022). Activation of STING Signaling. The STING signaling may be activated by either the administration of cGAMP (a cyclic di‐nucleotide, enzymatically synthesized by a cytoplasmic DNA sensor, also known as cGAS) or another non‐canonical STING agonist depicted in Figure 8 (Kumari et al. 2024). Rationally, chimeric exosomes loaded with STING agonists have been successfully developed, as shown in Figure 9 (Guo et al. 2025).

## 6 Rationale for a Dual‐Function Lipid Nanovector

Rapid CTL responses against primary brain tumors are often insufficient to control tumor progression and improve patient survival. Therapeutic strategies, such as immune checkpoint inhibitors (ICIs) that reinvigorate exhausted CTLs, have demonstrated efficacy in glioblastoma (GBM) models but have provided only marginal clinical benefit. These results suggest that the GBM microenvironment and/or treatment‐mediated effects on tumor biology can inhibit secondary AT immune responses capable of eliciting a response (Zou 2025).

**Figure 10:**
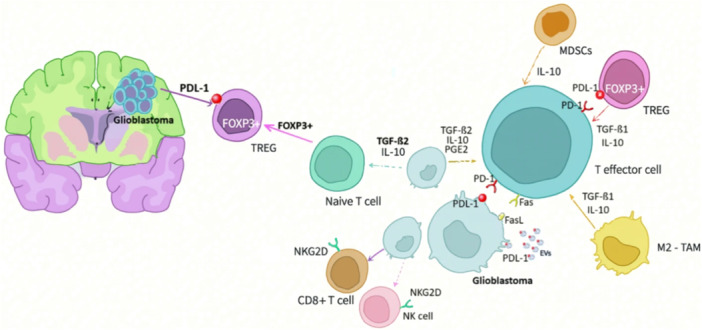
Depiction of the immunosuppressive microenvironment established by glioblastomas. MDSCs refer to myeloid‐derived suppressor cells, and NK to natural killer cells (Nourizadeh et al. 2025). Copyright Discov. Onc. 2025, 16, 1952. Diagram illustrating the immunosuppressive microenvironment created by glioblastomas. It shows interactions among various cells, including T regulatory cells, myeloid‐derived suppressor cells, and natural killer cells, with labels for PDL‐1, FOXP3, and NKG2D. The glioblastoma is central, with arrows indicating signaling pathways like TGF‐β and IL‐10.

Glioblastoma is maintained by a heterogeneous population of cancer stem cells (CSCs) that evade antitumor immunity and generate treatment‐resistant tumor relapse. Strategies to block tumor antigen (Ag) presentation and inhibit CTL priming in secondary or tertiary lymphoid organs have been reported. However, the involvement of these intratumoral and systemic immunosuppressive mechanisms after intratumoral therapy remains elusive (Chen, Peng, et al. 2023). Comprehensive investigations aimed at addressing the barriers to functional antitumor immunity and the true effectiveness of combined interventions should be conducted to develop novel approaches that could augment or improve immunotherapies, as depicted in Figure 10 (Nourizadeh et al. 2025).

**Figure 11:**
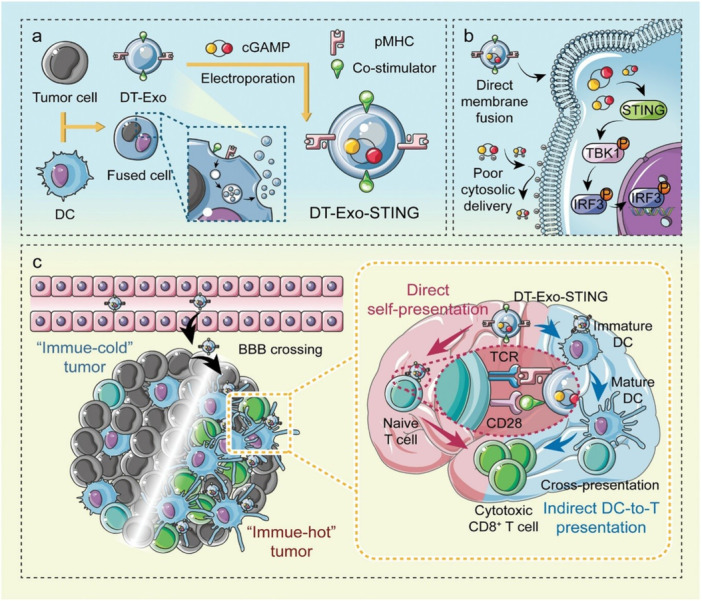
Schematic illustration of the DT‐Exo‐STING nanovaccine for glioblastoma immunotherapy. Design and manufacture of the DT‐Exo‐STING nanovaccine. b) Diagram illustrating the operational mechanism of this chimeric exosome‐mediated delivery approach in STING activation. c) Mechanism of DT‐Exo‐STING–induced antineoplastic immunoresponse in an intracranial glioblastoma mouse model. P denotes phosphorylation; TBK1 refers to TANK‐binding kinase 1; IRF3 signifies interferon regulatory factor 3 (Bao 2024). Copyright Advanced Science 2024, 11, 6, 2306336. Schematic illustration of the DT‐Exo‐STING nanovaccine for glioblastoma immunotherapy. Panel (a) illustrates the construction and preparation of the nanovaccine by electroporation of T‐PD‐Exos into DCs to generate DT‐Exo‐STING. Panel (b) shows how the action is exerted, meaning by membrane fusion and STING activation. Panel (c) immune response mechanism “immune‐cold” tumors transform into “immune‐hot” tumors through direct self‐presentation to naive T cells as well as indirect DC‐to‐T cell presentation, resulting in CD8^+^ T cell cytotoxic activation. The main agents are cGAMP, pMHC, and co‐stimulators.

To improve GBM treatment, a nanovector was developed to induce STING activation and polarize M1 microglia. STING activation elicits a robust type I interferon and pro‐inflammatory cytokine program in GBM tumors, promoting functional T cell infiltration and priming responses, as diagrammed in Figure 11 (Bao 2024). In preclinical models, the programmable cellular delivery of STING agonists directly to tumor GBM cells proved highly effective but, so far, has failed to show analogous therapeutic benefit in STING‐inactive tumors (Berger et al. 2022). Combination strategies that induce immunogenic cell death can expand the therapeutic profile of STING stimulation and represent an attractive approach for developing agents that may overcome prevalent immune escape mechanisms (Salvato and Marchini 2024). This dual‐function concept has gained support in various tumor models, and it appears that STING activation not only enhances interferon‐dependent antitumor immunity but can also drive macrophages residing within tumors towards a more inflammatory M1‐like phenotype, thus providing strong conceptual foundations for the application of such a combinatorial strategy to glioblastoma (Li, Yuan, et al. 2026; Zhang 2026).

### 6.1 Conceptual Framework: Simultaneous STING Activation and M1 Polarization

The rationale for concurrently inducing STING activation and M1‐like microglial/macrophage polarization is the complementary nature of these processes in converting a non‐immunogenic tumor colony into one that is immunogenic (Sun 2026). Activation of STING in antigen‐presenting and myeloid cells supports type I interferon‐ and inflammatory cytokine‐mediated enhancement of dendritic‐cell maturation and the priming and recruitment of CTLs (Li, Kang, et al. 2025). In parallel, this mechanism drives the polarization of tumor‐associated microglia/macrophages toward an M1‐like phenotype characterized by increased secretion of pro‐inflammatory mediators (TNF‐α, IL‐12, and IL‐6), improved antigen presentation, and enhanced phagocytic and tumoricidal activity (Winge et al. 2023). These two processes are mechanistically linked, rather than simply additive. Specifically, STING signaling can directly reprogram TAMs from an M2‐like, tumor‐promoting state to an M1‐like, antitumor phenotype in a host‐STING‐dependent manner, overcoming innate immune suppression and enhancing downstream adaptive immune activation (Wang, Bergholz, et al. 2022; Bi et al. 2026). In non‐glioma tumor models, STING agonism has been shown to enhance the M1/M2 macrophage ratio, reinforce type I interferon responses, and synergize with other anticancer therapies by restoring inflammatory immune crosstalk in the tumor microenvironment (Nourizadeh et al. 2025).

The relevance of this conceptual framework for glioblastoma is particularly important since the tumor is defined by intense myeloid‐cell‐based immunosuppression and a high frequency of M2‐like microglia/macrophages (Horta et al. 2025). A dual‐function nanovector that can deliver a STING agonist alongside an M1‐polarizing cue would thus address two of the main bottlenecks in systemic tumor therapy: poor innate immune activation and tumor‐promoting myeloid cell infiltration. Such designs are presumed to enhance local inflammatory signaling, antigen presentation, and T‐cell infiltration while also being more conducive to prolonged antitumor immunity (Erduran 2024; Youness et al. 2023). Therefore, the value of a bifunctional lipid nanovector lies not only in its utility to co‐deliver two cargos but also in its ability to coordinate immune conversion of the GBM‐M. Integration of STING‐driven innate sensing with active instruction of microglia/macrophages toward an M1‐like state effectively combines biologically redundant principles that can be harnessed for next‐generation GBM immunotherapy (Feng 2026; Pavani et al. 2026).

### 6.2 Potential Synergistic Mechanisms

Inhibition of the STING pathway in murine models suppresses type I interferons (IFN‐I) and proinflammatory cytokines, which are associated with DC maturation and antitumor effector cytotoxic T‐lymphocyte (CTL) responses that are known to play a substantial role in tumor immunity (Li, Zhao, et al. 2024). A modest enhancement of these responses could yield therapeutic benefit. In a melanoma model, STING agonists can potentiate immune responses elicited by tumor‐associated antigens (TAA) or neoantigens. The combination of a STING agonist with TAA‐encoding mRNA and poly(I:C) coated onto a lipid nanovector increases infiltration of effector memory CD8^+^ T cells and prevents tumor growth (Chen, Xu, et al. 2024). STING agonists also increase the recruitment of M1 macrophages and cytotoxic T cells as well as reprogram the tumor immune microenvironment across various tumors (Zhang 2023). In glioblastoma, the therapeutic benefit of STING pathway activation is route‐dependent, with local injection into the tumor or striatum being more effective in comparison to systemic delivery (Garland et al. 2022). The co‐encapsulation of a STING agonist and an RNA interference (RNAi) sequence targeting HRR (homologous recombination repair [HRR]) genes in a lipid‐based nanovector alters the tumor immune landscape toward an inflamed phenotype and improves response to immune checkpoint blockade (Peng et al. 2025). These dual‐function nanovectors also promote microglia polarization toward the M1 phenotype. They also induce microglial M1 polarization, in addition to their dual drug‐delivery ability (Wu 2025). Inhibition of receptor signaling for IFN‐αβ or injection of an M2‐phenotype–targeting agent diminished M1 polarization, suggesting that STING mediates M1 microglial reprogramming. Such a strategy highlights the value of a dual‐pronged lipid‐scaffold approach that simultaneously triggers STING activity and M1‐like differentiation (Malik et al. 2024).

## 7 Design Considerations for a Dual‐Function Nanovector

Lipid‐based nanovectors provide a promising platform for glioblastoma therapy (Huang 2023) and, when modified with a thermo‐magnetic trigger, enable both controllable cargo release and selective BBB penetration. Different lipid core structures can bind various effector molecules, including small molecules, proteins, nucleic acids, and particles (Pucci et al. 2020). Nanoformulations containing a magnetic payload react to an external magnetic field at physiological temperature and release their contents when heated, allowing for the simultaneous release of several agents at the desired location. Thermal activation also facilitates BBB transport and improves targeted delivery across the glioblastoma‐associated barrier (Wu et al. 2023; Amaral 2021).

The cGAS–STING pathway is important for anticancer immunity and is an attractive target for glioblastoma immunotherapy (Zhou et al. 2023). cGAS recognizes double‐stranded DNA released from dead cells, invading pathogens, or intracellular defects, generating cyclic GMP‐AMP (cGAMP) that activates STING (Bao 2024). Upon STING activation, they type I IFNs and other pro‐inflammatory cytokines through the transcription factors IFN regulatory factor 3 and NF‐κB. These elements facilitate DC maturation, direct tumor antigen presentation, and CD8 + T‐cell priming, thereby supporting antitumor immunity by activated T cells targeting cancer cells (directly) or by recruiting NK cells into the microenvironment (Zhang, He, et al. 2025; Nerdinger et al. 2025). Preclinical studies have indicated that STING agonists protect against glioblastoma growth in syngeneic rodent models (Najem et al. 2024). Antitumor responses are also enhanced by dual blockade of programmed cell death ligand 1 (PD‐L1) and CTL‐associated protein 4 (CTLA‐4) checkpoints (Rodriguez et al. 2024). The direct delivery of cGAMP into the glioblastoma tumor nests significantly enhances STING activation and type I interferon secretion within the tumor microenvironment. These findings demonstrate the promise of cGAS–STING‐targeted modalities for treating glioblastoma (Guo et al. 2025).

Emerging evidence highlights the immune‐evasive nature of the GBM‐M, a formidable barrier to effective immunotherapy (Wang, Yu, et al 2025). Microglia are the main innate immune effector cells in the central nervous system. They can switch between classical M1 and alternative M2 polarization states dynamically and reversibly. M2 microglia are the predominant phenotypic profile in glioblastoma ME and are known to contribute to tumor growth by promoting angiogenesis, enhancing tumor cell invasion, and inducing immunosuppression (Kuntzel and Bagnard 2022; Ren et al. 2023). A few studies have discovered candidates for the selective reprogramming of M2 microglia to M1, including adjuvants (TLR, CPG, and STING), inflammatory cytokines (TNF‐α, IL‐1β), and epigenetic inhibitors, indicating that we can preferentially guide a shift towards an M1 profile for these innate immune cells (Zhang, He, et al. 2025). Given microglia's role in glioblastoma progression, strategies targeting M2‐to‐M1 polarization require careful attention to achieve significant therapeutic effects as adjuvant therapy (Naik et al. 2025; Nusraty et al. 2024).

Inducing M1 microglial polarization represents a promising approach for glioblastoma immunotherapy. The highly successful establishment of therapeutic strategies aimed at promoting M1 microglial polarization, however, calls for the simultaneous delivery of STING agonists and M1‐inducing molecules (Battaglini et al. 2024; Wang, Guo, et al. 2024). A dual‐functional lipid nanovector that would activate STING and induce M1 polarization was expected to be an ideal immune potentiator to overcome several barriers to GBM immunotherapy and significantly improve therapeutic efficacy (Cha et al. 2025). This strategy enables the use of STING agonists and M1‐inducing factors in various combinations, depending on their available effects, providing flexibility in selecting molecules and increasing the number of candidates. In line with the foregoing, the following aspects are suggested for designing an amphiphilic lipid‐based nanovector with a dual task (Mu et al. 2024; Pesce et al. 2025).

A dual‐function lipid‐based nanovector must satisfy several important design criteria, including cGAS/STING‐targeted cargo selection, lipid composition, and thermo‐magnetic control of release and targeting (Ying et al. 2024). Dual‐function cargo candidates include cGAS/STING agonists, STING‐targeted RNAi, M1‐polarization target genes, and S/MAR or self‐amplifying mRNAs encoding the same payload (Tankov et al. 2024; Zhang 2023). Where an activator of STING, for example, cGAMP, ISD, or 3HP, is used as primary cargo, the selection of one or more unmodified mRNAs as a further secondary payload is likewise a preferred auxiliary alternative embodiment (Garland et al. 2022). Two other such second‐cargo candidates with potential include TLR 3 ligand Poly(I:C), TLR4 ligands LPS and MPLA, Adenosine A3 receptor (A3AR) agonist, Epigenetic inhibitors (Histone Deacetylases, Methyl Transferases, etc.), and other adjuvants (Zhang, He, et al. 2025; Mukherjee et al. 2023). Various delivery approaches, including encapsulation, co‐formulation, and surface adsorption, are amenable to lipid carriers for a second payload. Core‐shell lipid nanoparticles enable combined delivery of cGAMP and a TLR3‐ligand cRNA in numerous formats (Hegde et al. 2022; Musielak and Krajka‐Kuźniak 2025).

The chosen lipid formulation affects composition, charge, size, stability, release kinetics, and other important factors (Musielak and Krajka‐Kuźniak 2025). To optimize delivery to the central nervous system (CNS), the following criteria must be met: a net positive charge to improve interaction with the negatively charged cell membrane; a moderate size (60–120 nm) to balance a large hydrodynamic radius and nanocarrier clearance; structural stability against lipolysis and leakage during storage and physiological conditions; and sufficient membrane‐remodeling ability to ensure microglial engagement and trigger release upon contact with either the plasma membrane or the endo/lysosomal membrane. Selection of an appropriate lipid composition thus represents a central design consideration for achieving robust central nervous system‐targeted delivery (Jnaidi et al. 2020; Susa et al. 2024).

At least three interdependent strategies enable thermomagnetic triggering of dual‐function applications (Ceccarelli et al. 2025). Temperature‐response phase transformation integrated with magnetic‐field‐responsive heating is the most mature thermo‐magnetic trigger mechanism to date, and other candidates, that is, ferromagnetic particle loading, magnet‐acoustic joint stimulation, and magneto‐chemical reaction, are also promising additions that can complement temperature feedback (Liao et al. 2025). Immediately relevant melting temperature ranges are 37°C–41°C and 43°C–45°C, which define which materials may be used, similarly to materials with specific properties. Various approaches enhance the passive BBB permeability of dual‐targeting platforms while avoiding nonspecific accumulation in the normal surrounding tissue (Sarmah et al. 2025).

Assignment of this dual‐keyed system to glioblastoma remains an essential task (Wang, Sun, et al. 2025). Local and systemic delivery of STING‐activating payloads. While systemic delivery of STING‐activating cargoes can generate a strong anti‐tumoral immune response on its own, this suggests that the first barrier may be bypassed without specific targeting (Zhang 2023). Investigation of active targeting moieties targeting different markers of glioblastoma should be considered to expand the number of strategies used not only to minimize off‐target effects of dual‐function systems but also in the CNS and peripheral nervous system (Shen 2024).

### 7.1 Cargo Selection and Loading Strategies

Lipid‐based nanovectors can deliver a wide variety of cargos using easily adjustable methods. Thermosensitive and magnetic nanoparticles can be used advantageously to formulate a thermosensitive material (Gupta et al. 2023; Pesce et al. 2025). A limited set of biocompatible lipids that are compatible with complex composition mixtures and formulation processes can be used. In an externally multi‐magnetic‐field switchable setting, heating at a defined threshold concentration was used to physically induce micellization and payload release, allowing cargo distribution, bypassing the BBB, and activating microglia (Horta et al. 2025).

Designed nucleic acids can upregulate endogenous STING expression, thereby facilitating the delivery of lipid‐mediated exogenous cGAMP or cGAMP–protein nanoparticles (Fu et al. 2025; Qiao et al. 2025). Artificial mRNA constructs (lipid nanoparticle‐formulated) carrying ISGs, pattern‐recognition receptors, or STING‐, pSTING‐, and TCF1‐activating effectors, including fifth‐generation synesthete, have previously been developed (Muslimov et al. 2023). Pathogen‐ or damage‐associated molecular patterns, either as small molecules or via in vivo delivery by genetically modified strains, stimulate cGAS (or bacterial cGAMP). TLR‐3, TLR‐4, or TLR‐7 agonists, administered concurrently or via oncolytic viruses, provoke stimulation of STING and type‐1 helper T cell responses (Garland et al. 2022).

Beyond STING, systems that elicit dual cGAS–STING and NF‐κB cascades permit simultaneous engagement of cytosolic and membrane‐tethered pattern‐recognition receptors (Low et al. 2024; Islam et al. 2024). TAAs, formulated as protein–nucleic‐acid cocktails in polymeric microcapsules, activate STING and promote cross‐presentation without prior electrotransfer. Different platforms make it easier to send stimulatory signals in a precise, organized manner (Mu et al. 2024). Table 4 maps specific combinations of STING agonists plus M1‐polarizing agents to their synergistic mechanisms.

**Table 4:** Dual‐function Cargo combinations and their mechanistic roles.

Sr. No.	Cargo 1 (STING agonist)	Cargo 2 (M1 inducer/innate immune adjuvant)	Mechanistic synergy	Immune outcome (in TME/microglia/macrophages)	Delivery system	Reference
1	CDN (e.g., 2′3′ cGAMP or other cyclic dinucleotide)	TLR7/8 agonist (e.g., imidazoquinoline analog)	Simultaneous activation of cytosolic DNA sensing STING pathway (type I IFN, NF‐κB) + endosomal TLR7/8 pathway (pro‐inflammatory cytokines, NF‐κB); amplified innate activation.	Strong M1/microglia‐like polarization, enhanced cytokine/chemokine milieu, improved antigen presentation	Dual‐agonist nanoparticles or nano‐vaccines encapsulating both agonists to co‐deliver them to the same cell. This “multi‐adjuvant” approach improves APC activation and cross priming.	(Bhatnagar et al. (2022))
2	CDN (e.g., 2′3′ cGAMP) in endosomolytic/polymeric carrier	Tumor antigen peptide or neoantigen	STING activation + efficient antigen delivery/enhanced cross presentation by APCs	M1‐like macrophages/microglia + robust CD8^+^ T cell priming against tumor antigens	Nano‐vaccine (e.g., polymersomes) that release STING agonist intracellularly and present antigen to dendritic cells/macrophages. Demonstrated improved anti‐tumor immunity in preclinical cancer models	(Chen, Xu, et al. (2024))
3	CDN (STING agonist)	Damage‐inducing therapy (e.g., radiotherapy or chemotherapeutic platinum drug)	Damage therapy causes tumor cell death and the release of tumor antigens and DAMPs; STING agonists activate APCs to sense these; synergy between cell death‐associated antigen release and STING‐driven innate immune activation.	M1/macrophage microglia polarization, enhanced phagocytosis of dying tumor cells, improved antigen presentation, increased CD8^+^ T cell infiltration and tumor control	Combining STING pathway modulators with radiotherapy or chemotherapy has been proposed to improve response by converting immunologically “cold” tumors into “hot” tumors. Preclinical rationale supports this for resistant tumors, including glioblastoma.	(Sun, Fu, et al. (2025))
4	CDN (STING agonist)	TLR2 agonist (or TLR2 ligand + other innate cues)	STING activation primes type I IFN + NF‐κB; TLR2 provides additional NF‐κB/pro‐inflammatory signaling, possibly enhancing macrophage activation beyond STING alone	Enhanced M1 polarization, increased pro‐inflammatory cytokines, and augmented tumor inhibitory microenvironment	This combination has recently been shown to improve antitumor efficacy compared with STING agonist alone in murine models. Could be adapted to microglia/macrophage in the GBM context	(Song et al. (2025))
5	CDN (STING agonist)	Immune modulatory metal ions/metal ion adjuvant (e.g., specific divalent metal ions that enhance cGAS‐STING activation)	Metal ions may stabilize cyclic dinucleotides or enhance cGAS/STING signaling; together with an STING agonist, this boosts type I IFN and downstream innate responses.	Potent macrophage/microglia activation, elevated IFN‐stimulated genes (ISG), enhanced local inflammation, and immune cell recruitment.	An emerging concept is using metal‐ion cofactors to potentiate STING signaling, which may allow lower doses of CDNs. Helpful in designing lipid‐based nanovectors with metal components	(Li, Xu, et al. (2025))
6	CDN (STING agonist) in a nanocarrier	Nanoparticle formulation that itself promotes phagocytosis (e.g., “eat‐me” signals, targeting moieties to microglia/macrophages) + optionally TLR ligand or adjuvant	Nanocarrier increases uptake by microglia/macrophages, delivering the STING agonist directly into the phagocyte, potentiating STING activation in these cells rather than tumor cells, leading to microglia/macrophage reprogramming.	Direct M1 polarization of microglia/macrophages in TME; enhanced antigen uptake and presentation; reshaping suppressive TME into an inflammatory, tumor‐hostile milieu	This is a rational design, especially for brain tumors like glioblastoma, that targets microglia/macrophages rather than tumor cells. The general feasibility of STING agonist nanocarriers has been well reviewed.	(Chen, Xu, et al. (2024))
7	CDN (STING agonist) encapsulated in biodegradable nanoparticles (e.g., PACA or PGA‐based)	TLR agonist (e.g., TLR9 agonist such as CpG‐ODN, or other TLR ligand) + tumor antigen	Co‐delivery ensures the same APC or macrophage receives both STING and TLR signals plus tumor antigen; synergistic upregulation of costimulatory molecules, cytokines, antigen presentation, and cross‐priming	M1/M1‐like macrophage polarization, dendritic cell activation, enhanced CD8^+^ T cell responses, “cold‐to‐hot” TME conversion	Polymeric nanoparticles offer tunable biodegradability and can reduce systemic toxicity; they are widely considered promising for cancers, including those resistant to treatment, such as glioblastoma.	(Huang (2022))
8	Polymer–drug conjugate releasing STING agonist (e.g., polymer–CDN conjugate)	Hypoxia‐responsive or ROS‐responsive chemotherapeutic/cytotoxic agent (or other stress‐inducing drug)	Cytotoxic drug induces tumor cell stress/death; release of tumor antigens and DAMPs; polymer releases an STING agonist to nearby APCs; synergy of antigen release + innate stimulation.	M1 type macrophage/microglia activation, increased antigen uptake, cross presentation, adaptive T cell activation	Recent work shows polymer‐STING conjugates improve tumor accumulation and reduce systemic toxicity. Could be combined with cytotoxic stress‐inducing agents for maximal immunogenicity	(Sheehy et al. (2024))

### 7.2 Lipid Composition and Physicochemical Properties

To promote effective delivery of STING agonists to glioblastoma and potentiate antitumor immunity via tumor‐targeted M1 microglia polarization, a lipid nanovector was designed to combine the cGAS‐STING pathway activator ADU‐S100, a siRNA targeting the immunosuppressive cytokine CCL2, and a model mRNA encoding the immunogenic tumor antigen ovalbumen (OVA) (Fu et al. 2025; Xu and Xiong 2024). The agent, assembled from a cationic lipid matrix, anionic poly(ethylene glycol)‐lipid, and OVA mRNA, exhibited a positive surface charge, ensured rapid cellular internalization, and enabled cytosolic delivery of large RNA cargo (Musielak and Krajka‐Kuźniak 2025). The lipid formulation supported high ADU‐S100 loading, a release profile that matched intracellular trafficking rates, and endosomal escape (Guo et al. 2025). Co‐delivery of ADU‐S100 with either CCL2 siRNA or OVA mRNA enhanced M1 polarization and intratumoral interferon‐γ (IFN‐γ) response compared to either agent alone, and the dual‐function formulation improved therapeutic outcomes against OVA‐expressing GL261 tumors in syngeneic mice (Mahajan et al. 2025; Ye et al. 2019).

The basic mass constituent of cationic lipid nanocarriers determines their physicochemical characteristics and their interactions with cells (Sedky, Abdel‐Kader, et al. 2023). Screening an in‐house lipid library allows tuning the transport vesicle for extracellular stability, brain entry, cytosolic translocation, endosomal release, and selective glioblastoma targeting (Garg et al. 2022; Wafik Nabih et al. 2025; Hegde et al. 2022). The formulations that were subsequently investigated included a cationic lipid, neutral co‐lipid or helper lipid, anionic lipid, and poly(ethylene glycol)‐lipid conjugate (PEG‐lipid) complexed to a targeting ligand. These lipid structures facilitate the encapsulation of nucleic acids of different sizes and are compatible with various loading methods, such as lipid‐film hydration (Pucci et al. 2020).

### 7.3 Thermo‐Magnetic Control and Bbb Penetration

Brain tumors are still generally highly malignant cancers, and GBM is the most common primary brain tumor in adults. The invasive and rapid growth of the tumor and associated resistance to therapy lead to a dismal prognosis. Patients usually survive a median of 15 months, and fewer than 10% live beyond 5 years. ICIs have demonstrated favorable outcomes in select solid tumors, but in GBM, results have been negative, suggesting there are novel avenues to pursue. This immunosuppressive environment within GBM represents a hurdle to immunotherapies (Lee 2021; Eckert et al. 2025).

**Figure 12:**
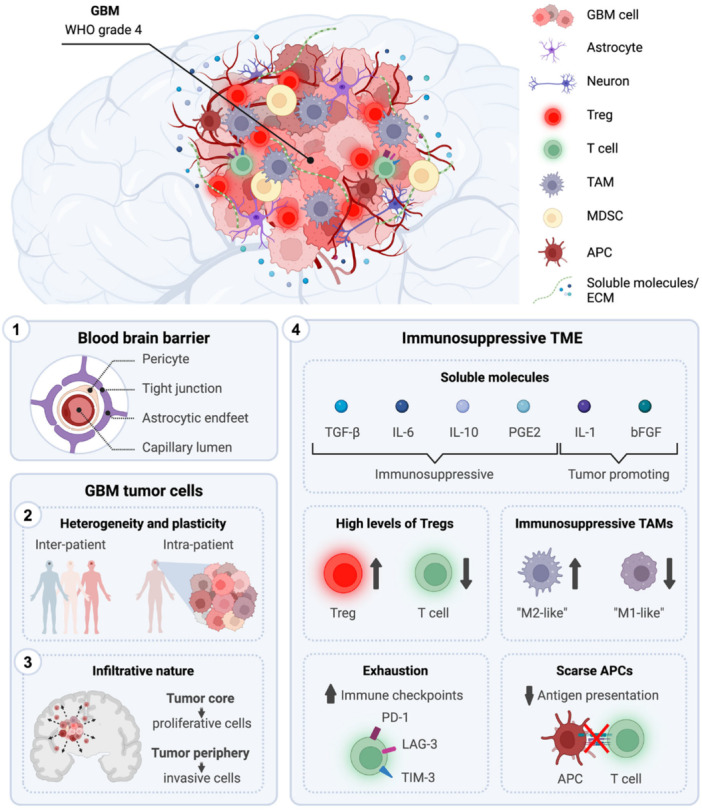
Therapeutic obstacles are present in the treatment of glioblastoma multiforme (GBM). APC, refers to antigen‐presenting cell; bFGF denotes basic fibroblast growth factor; ECM signifies extracellular matrix; GBM stands for glioblastoma; IL, indicates interleukin; LAG‐3 represents lymphocyte‐activation gene 3; MDSC means myeloid‐derived suppressor cells; PD‐1 is programmed cell death protein 1; PGE2 refers to prostaglandin E2; TAM, denotes tumor‐associated microglia and macrophages. TGF‐β, transforming growth factor‐β; TIM‐3, T‐cell immunoglobulin and mucin domain; TME, tumor microenvironment; Treg, regulatory T cell; WHO, World Health Organization. The picture delineates the unique attributes of GBM (WHO grade 4) that are recognized as impediments to the advancement of efficacious anti‐tumor therapy. These factors comprise (1) an anatomical region protected by the blood‐brain barrier, (2) intra‐ and inter‐patient tumor heterogeneity, (3) infiltrative characteristics, and (4) a markedly immunosuppressive tumor microenvironment. The latter demonstrates the presence of GBM‐induced cytokines with both immunosuppressive and tumor‐enhancing properties, including immunosuppressive cell types such as Tregs and M1‐like TAMs, as well as elevated exhaustion markers. Moreover, GBMs intentionally downregulate antigen‐processing and presentation molecules to evade T cell activation (Salvato and Marchini 2024). Copyright Cancers 2024, 16, 7, 1276. The figure shows therapeutic barriers to GBM treatment options for glioblastoma multiforme (GBM) that do not significantly extend patient survival. The middle image depicts a GBM tumor with different cell types identified, including GBM cells, astrocytes, neurons, T cells, and others. Four main panels describe challenges: (1) Blood‐brain barrier features, such as pericytes and astrocytic end feet; (2) GBM‐specific cell heterogeneity and plasticity: this includes intra‐patient and inter‐patient diversity between tumors; (3) Highly invasive nature of tumor cells; (4) Immunotolerant TME with immune cell contacts and soluble mediators. Crucial factors include high frequencies of regulatory T cells, an inhibitory tumor‐associated macrophage environment, and exhausted immune checkpoints.

The GBM immunosuppressive milieu is characterized by a diverse array of immunosuppressive cells, including regulatory T cells (Tregs), myeloid‐derived suppressor cells, and M2‐polarized microglia (Beola et al. 2023). Tregs are the first immune cells to enter GBM and exert strong immunosuppressive effects. While Tregs in other tumors exhibit a cancer‐specific phenotype, Tregs in GBM retain a non‐cancer‐specific transcriptional profile. Current FDA‐approved strategies do not directly address GBM‐specific immunosuppression. Infiltrated effector T cells exhibit reduced cytotoxicity (Pucci et al. 2020). Effector T cells need to be expanded against antigens associated with GBM, and progress has been made in developing GBM vaccine strategies; however, the treatment remains limited. A new synthetic biology circuit driven to make tumor cells express an engineered form of IL‐12 has demonstrated a 15‐fold reduction in tumor volume under pure preclinical conditions, as reported in (Figure 12) (Salvato and Marchini 2024; Vo et al. 2025).

Although significant progress has been made in solid tumor immunotherapy, the BBB remains a significant challenge in treating GBM. Delivering immunotherapy directly to the brain would be warranted given preclinical successes across multiple approaches; yet this work has not been pursued in earnest, perhaps indicating that a successful example of therapy targeting one organ would spur such important work (Salvato and Marchini 2024; Lechpammer et al. 2022). The BBB structure serves as a crucial barrier to the central nervous system, limiting the availability of crucial therapeutic drugs. The anatomy and biology of the BBB, strategies for drug delivery to the sites of action, and methods for circumventing this challenge are described here (Wu et al. 2023; Susa et al. 2024). Finally, perspectives on dual‐acting vector design are anticipated to remain central to advancing this clinically relevant strategy across CNS tumors (Duan et al. 2024).

### 7.4 Targeting Ligands and Specificity

Lipid‐based nanovectors have recently emerged as promising tools for delivering drugs, RNAs, proteins, and other molecules to treat various cancers, including glioblastoma. Nanovectors are advantageous owing to their biocompatibility, ease of preparation, flexible design, and tunable drug loading (Gupta et al. 2023; Miao et al. 2023). Generally, depending on the type of lipid amphiphiles that self‐assemble into particles in vivo, nanovectors can load and encapsulate diverse chemotherapeutic drugs, nucleic acids, peptides, genes, or immunomodulatory adjuvants for codelivery (Hegde et al. 2022; Musielak and Krajka‐Kuźniak 2025). The release of cargo can be triggered by applying various stimuli, such as thermal, redox, chemical, and magnetic fields, to these delivery vehicles (Tapeinos et al. 2019; Ismail et al. 2024). These carriers, together with selected payloads, can prolong action profiles, optimize pharmacokinetics, and deliver drugs selectively to non‐target sites, thereby reducing off‐target effects and improving efficacy after drug administration to the patient (Rahman et al. 2025).

Integral to their development and evaluation are versatile thermosensitive lipids that melt at physiological temperature (37°C) and become fluid above the body temperature (°C) (Musielak and Krajka‐Kuźniak 2025; Sedky, Braoudaki, et al. 2023). These lipids form solid nanovectors at room temperature. However, transition to a liquid state upon warming with an external stimulus, permitting payload release below the melting point either upon arrival at a target site or prior to infusion (Garg et al. 2022). Furthermore, the inclusion of magnetic nanoparticles into the lipid formulation adds functionality. The application of an external magnetic field can cause heating and drug release, or it can be used to attach nanoparticles to a targeting ligand for in situ delivery of payloads (Khot et al. 2024). These strategies are quite promising for several biomedical applications, including anti‐cancer therapy, with inherent advantages for central nervous system (CNS) tumors (Jindal et al. 2025).

**Figure 13:**
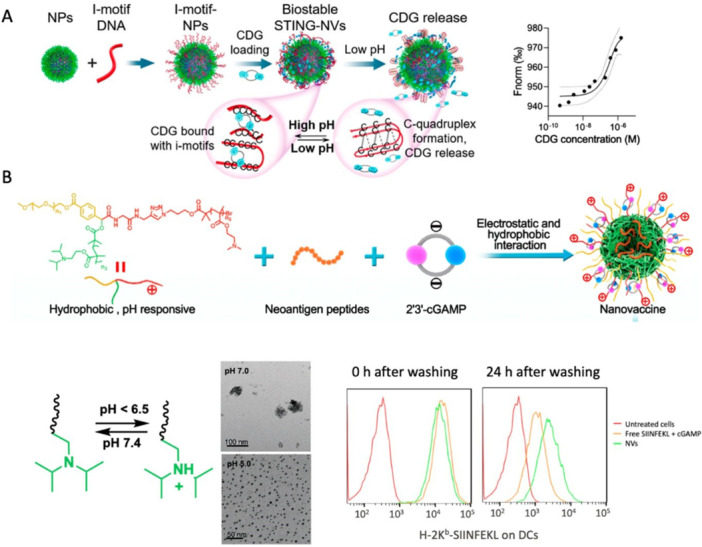
Creation of STING agonist‐based nanovectors for cancer immunotherapy. (A) Design of STING‐NVs utilizing pH‐responsive DNA i‐motif‐functionalized PEG‐*b*‐PLA nanoparticles for the loading of CDG by hydrogen bonding. These STING‐NVs effectively encapsulated CDG through hydrogen bonding, namely G:C base pairing. Under acidic circumstances, CDG dissociated from i‐motifs due to the formation of C‐quadruplexes from i‐motifs via protonated C:C^+^ base‐pair formation. The binding affinity (*K*_d_) of CDG with NPs, which serves as the driving force for CDG loading in these nanoparticles, was quantified at 47 × 10^−9^ M using microscale thermophoresis (MST). (B) pH‐responsive polymeric nanovesicles for the simultaneous delivery of cGAMP and tumor neoantigen peptides via electrostatic and hydrophobic interactions, respectively. The pH‐responsive PDPA moiety was protonated in acidic environments, such as endolysosomes within APCs, to facilitate NV dissociation and vaccine release from nanoparticles. The co‐delivery of cGAMP and SIINFEKL peptide‐maintained antigen presentation even after the removal of exogenous vaccinations, as demonstrated by flow cytometric analysis utilizing a dye‐labeled antibody for the SIINFEKL/H‐2K^b^ complexes (H‐2K^b^ is a subtype of murine MHC‐I). The NVs enhanced the neoantigen‐specific T cell responses, leading to effective tumor immunotherapy (Zhou et al. 2023). Copyright Acc Chem Res. 2023, 56, 21, 2933–2943. Schematic flow chart of the preparation of STING agonist‐based nanovectors for cancer immunotherapy. The structure of STING‐NVs formulated by pH‐responsive DNA i‐motif‐modified PEG‐b‐PLA nanoparticles for CDG loading is presented in panel (A). These include the processes of CDG conjugation, biostable STING‐NV generation, and CDG release at low pH. CDG concentration is plotted against fluorescence on the graph. Panel (B) shows pH‐responsive polymeric nanovesicles for cGAMP and neoantigen peptide delivery, showing electrostatic and hydrophobic interactions. Further diagrams of chemical structures, pH conditions, and associated flow cytometry results for untreated cells compared with treated cell populations over time.

The strong anti‐cancer immunity: cGAS recognizes cytosolic double‐stranded DNA (dsDNA) from dying tumor cells or necrotic cells (Dong 2025). It mediates the synthesis of cGAMP, which, in turn, binds to and activates STING, as demonstrated in Figure 13 (Zhou et al. 2023). The cGAMP‐STING complex induces immune signaling that upregulates interferon (IFN)‐stimulus‐response genes and causes the synthesis of various effector molecules and cytokines, such as type I IFNs, interleukin (IL)‐12, and IL‐23 that mediate systemic antitumor immunity through dendritic‐cell maturation, T‐cell priming, and NK cell activation by both tumor endothelial cells and macrophages (Zhang, Li, et al. 2025). The cGAS‐STING pathway has been recognized as playing a key role in cancer immunotherapy, and efforts are being focused on small‐molecule STING agonists (Ye et al. 2019).

Several lipid‐based systems and bioengineered protein nanoparticles targeting tumor growth via the cGAS‐STING pathway were developed (Fu et al. 2025). Cyclic Dinucleotide STING Agonists as Cancer Therapeutics: In preclinical glioblastoma models, cyclic dinucleotide STING agonists delivered both intratumorally and systemically trigger cGAS (cGAMP) and STING signaling pathways in both malignant tumor cells and the tumor's immune microenvironment (Low et al. 2024). Anticancer effects have been reported in a dose‐dependent manner after treatment with such agonists, both alone and in combination with immune checkpoint blockade (Rodriguez et al. 2024).

The GBM‐M comprises diverse immune cells, such as pro‐inflammatory M1 macrophages, interferon‐γ (IFN‐γ) plus NK cells, and cytotoxic CD8^+^ T lymphocytes, and extensive gene set and differential gene‐expression analyses have demonstrated the critical roles of CTL infiltration and polarization of TAMs into the M1 phenotype in promoting glioblastoma antitumor immunity (Zhao et al. 2025; Tu et al. 2021). However, tumor‐intrinsic mechanisms, including aberrant cell‐intrinsic signaling pathways and epigenetic regulation, inhibit the cGAS‐STING pathway and CTL responses (Wang, Yu, et al. 2025). As a result, current strategies to invigorate anti‐tumoral immunity with STING agonists alone do not sufficiently address the multiple barriers posed by glioblastoma (Zhang 2023).

## 8 Preclinical Evaluation Paradigms

Preclinical evaluation of a dual‐modality lipid nanoparticle in the context of its validity for application as an allosteric inducer, which could promote antitumoral immunotherapy against glioblastoma via STING‐activation‐mediated microglial M1 polarization (Zhou et al. 2022). Three principal questions underpin this assessment: does the system successfully push M1 characteristics on glioblastoma‐related microglia (Menna et al. 2022); does this process induce anticancer immune responses of clinical relevance (Salvato and Marchini 2024); and how safe is the treatment, including potential adverse events on long‐term safety observations (Sipos et al. 2025). To address these problems, a correspondence between in vitro and in vivo studies using established GBM models in both immunocompetent and humanized systems is proposed (Thomas and Rahman 2025).

In vitro investigations using microglia–glioblastoma co‐cultures may demonstrate whether the dual‐function platform induces microglial M1 polarization, triggers pro‐inflammatory cytokine secretion, and elicits various cytotoxic mechanisms to treat glioblastoma, such as T‐effector‐cell responses and antibody‐dependent cell‐mediated cytotoxicity (Wang, Guo, et al. 2024; Ijaz et al. 2025). Together, these parameters provided quantitative metrics for the predicted pro‐immunogenic activity of a dual‐function approach.

Preclinical in vivo studies can be conducted using immunocompetent, syngeneic, or humanized glioblastoma models to assess therapeutic benefits and potential neurotoxicity (Zannikou et al. 2025). Preclinical endpoints include survival, tumor size, immunological parameters such as the presence of immune cells and their activation state, and safety readouts, including neuroinflammatory states and off‐target biological effects (Salvato and Marchini 2024). These criteria facilitate detailed testing of therapeutic efficacy, investigation of underlying mechanisms, and appraisal of potential safety risks (Bao 2024).

### 8.1 In Vitro Models: Microglia and Glioblastoma Co‐Cultures

Co‐culture models combining mouse glioblastoma cell lines with either mouse microglia or astrocytes have been utilized to monitor the effects of tumor–stroma interactions on Oncostatin M and IL‐1β expression (Hong et al. 2021). Likewise, human glioblastoma lines can be co‐cultured with human primary microglia or microglial‐like THP‐1 cells to investigate whether glioblastoma cells produce inhibitory factors that suppress IL‐1β expression (Chen et al. 2020). Table 5 aids in selecting standardized assays for immune response evaluation.

**Table 5:** In vitro assays for evaluating dual‐nanovector activity.

Sr. No.	Assay name	Cell types/models used	Readout(s)/measurement	Relevance to dual nanovector (STING + M1) strategy	Considerations	Reference
1	Co‐culture of nanovector‐activated DCs with T cells in 3D GBM spheroids	3D GBM spheroids + pre‐activated T cells	Tumor infiltration, T cell activation, tumor lysis in a 3D context	An advanced model to simulate T‐cell infiltration and killing in a tumor‐like environment	Use live imaging/fluorescent reporters for spatial‐temporal monitoring	(Hong et al. (2021))
2	Cytokine/Chemokine quantification (ELISA/Multiplex/Cytometric bead array)	Microglia (primary or cell line, e.g., BV2), macrophages, dendritic cells, possibly co‐cultured with tumor cells	Levels of pro‐inflammatory cytokines/chemokines (e.g., IFN‐β, TNF‐α, IL‐1β, IL‐6, CXCL10, IL‐12, etc.) in supernatant	Demonstrates functional activation of innate immune response via STING; M1 skewing; cytokine milieu critical for immunotherapy efficacy.	Multiplex or bead‐based flow cytometry can increase throughput; it is important to include controls (unstimulated, nanovector without STING agonist, LPS, or known STING agonist)	(Kaushik and Basu (2013))
3	Cross‐presentation assay (Model antigen: OVA)	DCs + OVA‐expressing tumor cells + OT‐I CD8^+^ T cells (mouse), or peptide‐pulsed human DCs + autologous CD8^+^ T cells	T cell proliferation (CFSE dilution), IFN‐γ secretion (ELISPOT or ELISA), cytotoxicity assay	Tests if the nanovector facilitates tumor antigen cross‐presentation to CD8^+^ T cells; crucial for antitumor T‐cell responses	Can use model system like OVA–OT‐I or real tumor antigens; include nanovector without STING for comparison	(Chen, Meng, et al. (2023))
4	ELISPOT for IFN‐γ/IL‐2/TNF‐α secreting T cells	DC^+^ T cell co‐culture or isolated CD8^+^/CD4^+^ T cells stimulated ex vivo	Spots representing cytokine‐producing T cells (per well)	Measures functional antigen‐specific T‐cell responses; beneficial for STING‐based vaccine‐like nanovector effects	Sensitive and quantitative; can be used for recall assays; use appropriate antigens (e.g., peptides or GBM lysates)	(Wang, Liu, et al. (2022))
5	Gene expression analysis (qRT‐PCR, RNA seq/bulk or single cell)	Microglia/macrophages (primary or cell line), dendritic cells; possibly mixed or co‐culture models with tumor cells	mRNA levels of type I IFNs (e.g., IFN β), interferon‐stimulated genes (ISGs), M1 markers (e.g., iNOS, IL 1β, IL 6), M2 markers (e.g., Arg1, IL 10), and other immunomodulators (e.g., MHC molecules)	Provides transcriptomic evidence of polarization (M1 vs. M2) and immune activation. Useful for early signaling changes and for comprehensive profiling	Can be complemented by RNA‐seq for broad profiling; in co‐culture models, it may reveal cross‐talk between tumor cells and microglia/macrophages	(Chen (2025))
6	Flow Cytometry (Surface/Intracellular Markers)	Microglia/macrophages/dendritic cells (possibly from rodents or human monocyte‐derived)	Surface markers (e.g., CD86, CD80, MHC II, CD206, CD163), intracellular markers (e.g., iNOS, cytokines), and possibly viability/apoptosis	Quantitative analysis of polarization state (M1 vs. M2), maturation status (in DCs), and phagocytic potential distinguishes subpopulations in mixed cultures or heterogeneous responses.	Multiparametric flow allows simultaneous readout of multiple markers, intracellular cytokine staining, or iNOS, depending on the availability of antibodies for the chosen species.	(Kaushik and Basu (2013))
7	Phagocytosis/Tumor cell clearance assay (Co culture + Fluorescence/Imaging/Flow)	Microglia or macrophages (primary or cell line) + glioma cells (e.g., GBM cell lines)	The percentage of macrophages/microglia that engulf tumor cells, or the number of tumor cells cleared, can be measured by fluorescent labeling and flow cytometry or microscopy.	Direct functional readout shows that not only are immune cells “polarized,” but they also engage in tumor cell clearance, which is especially relevant for a dual‐function nanovector combining STING agonism with phagocytosis, checkpoint blockade, or opsonization.	Useful when the nanovector also carries agents (e.g., anti‐CD47) or when testing enhanced phagocytosis + STING activation synergy; controls should include the nanovector without STING agonist, with and without co‐delivered agents.	(Zhou et al. (2022))
8	Polarization/Phenotyping via microglia functional assays (e.g., Nitric oxide production, iNOS expression, ROS, markers of activation)	Primary microglia or microglial cell lines; macrophages	Nitric oxide (NO) production; reactive oxygen species (ROS); expression of iNOS; morphological changes; other functional readouts	M1 microglia are often characterized by high iNOS/NO, ROS production, and distinct morphology; this gives additional functional validation beyond cytokines or markers	Combine with other readouts (cytokines, surface markers) for robust phenotype definition; consider time‐ and dose‐dependence; account for baseline activation in culture	(Maguire et al. (2022))
9	STING‐dependent reporter assays (e.g., IFN β promoter luciferase, ISG‐reporter cells)	Reporter cell lines (e.g., microglia or macrophage lines transduced with luciferase under IFN‐β or ISG promoter), or primary immune cells transfected with reporter constructs	Luciferase (or other reporter) activity indicating promoter activation	High‐sensitivity, quantitative assay for STING activation; good for screening nanovector formulations, dose‐response, kinetics	Reporter assay is fast, scalable, ideal for initial screening; must validate that reporter behaves similarly in relevant primary cells; consider transfection efficiency or stable cell line generation	(Mahajan et al. (2025))
10	T cell priming and activation assay (DC: T cell co‐culture)	DCs (nanovector‐primed) + nave CD4^+^ or CD8^+^ T cells	T cell activation markers (CD69, CD25), proliferation (CFSE), cytokines (IL‐2, IFN‐γ), cytolytic markers	Evaluates the generation of effector T cells by nanovector‐activated DCs, a critical link between the innate and adaptive immune responses	Aids in confirmation that immune activation correlates with T cell engagement; species‐matched systems are key	(Qiu et al. (2023))
11	Western blot/Immunoblot to assess triggering of the STING pathway	Microglia, macrophages, dendritic cells (in vitro)	Protein expression/phosphorylation of the components in the STING pathway (e.g., pSTING, p TBK1, p IRF3, NF‐κB p65)	Verifies the involvement of canonical STING signaling cascade in nanovector activation; mechanistic evidence that goes beyond cytokine readout	Make sure to do both time‐course and dose‐response; you may also want to blot for total versus phosphorylated protein levels and include loading controls	(Yang et al. (2024))

### 8.2 In Vivo Models: Immunocompetent and Humanized Systems

The review details the use of dual‐function lipid‐based nanovectors to trigger STING and repolarize M2 tumor‐associated microglia into an immunogenic M1 phenotype in glioblastoma (Battaglini et al. 2024). Immunotherapy is a novel approach in the management of glioblastoma, but immune escape can be exploited to promote tumor progression (Yu et al. 2023). It has been reported that glioblastoma hinders effector T‐cell activity (infiltration) and function by restricting antigen presentation and by activating exhaustion checkpoints on immune cells, which utilize a wire‐like architecture (Yaacoub et al. 2025). In addition, the GBM‐M is highly infiltrated by protumoral M2‐type microglia, leading to immunosuppression (Zhang, He, et al. 2025). While several approaches have been developed to boost immune responses against glioblastoma, the field lacks a strategy capable of overcoming concomitant multiple co‐inhibitory mechanisms or epigenetically reprogramming tumor‐associated microglia (Su et al. 2025; Tang et al. 2025).

cGAS–STING is an intracellular receptor that senses the cytosolic delivery of adenine or guanine dinucleotides and activates type I interferon (IFN) signaling to elicit immunity against many types of cancer (Zhang, Li, et al. 2025). In murine orthotopic glioblastoma models, STING is activated in proneural GBM, leading to reduced tumor growth and improved survival (Sun, Kang, et al. 2025). Thus, in the TME and secondary lymphoid organs, M1‐polarized microglia release pro‐inflammatory cytokines, enhance antigen presentation, and provide helper functions for effector T‐cell priming within tumors, thereby eliciting T‐cell infiltration and cytotoxicity (Ma 2021). M1‐polarizing agents mediate the recovery of anti‐tumor immunity in immunologic “cold” murine GBM models and can be combined with immune‐checkpoint inhibitors in “hot” tumors (Ijaz et al. 2024). Such delivery using the same platform has never been reported previously in the context of STING agonists and M1‐inducing agents (Battaglini et al. 2024). Table 6 provides an overview of model systems and endpoints used to assess therapy efficacy.

**Table 6:** Preclinical models and readouts for dual‐nanovector testing.

Sr. No.	Model	Species/host	Tumor type/configuration	Delivery route and example nanovector	Primary efficacy readouts	Mechanistic/immune readouts (relevant to STING + M1)	Reference
1	Brain‐targeting STING‐activating nano‐assembly	Mouse (immunocompetent)	GL261 orthotopic GBM, bilateral GBM model (primary + distant site)	Intravenous nano‐assembly TCe6@Cu/TP5 NPs (photosensitizer + copper + TP5); photodynamic activation	Tumor growth in primary and distant lesions, survival, and response at the distant non‐irradiated site	cGAS–STING activation (cGAS, STING, p‐TBK1, p‐IRF3), PD‐L1 downregulation, DC maturation, CD8^+^ T‐cell infiltration, cytokines (TNF‐α, IFN‐γ)	(Chen, Tian, et al. (2024); Nady et al. (2023))
2	HER2‐directed STING agonist ADC platform (non‐CNS, template for systemic targeting)	Mouse (immunocompetent and immunodeficient)	Multiple HER2^+^ xenograft and syngeneic models	Intravenous XMT‐2056 or related STING‐ADC constructs	Tumor regression after single or multiple doses, comparison versus free agonist, and tolerability	Tumor‐localized STING activation, reduced systemic cytokines versus free agonist, DC/T‐cell activation, macrophage repolarization	(Bukhalid et al. (2025))
3	Intratumoral STING agonist in spontaneous canine GBM	Dog (spontaneous glioma)	Spontaneous high‐grade glioma/GBM in client‐owned dogs	Stereotactic intratumoral injection of non‐CDN STING agonist IACS‐8779	MRI response (partial responses, stable disease), progression‐free and overall survival, clinical signs	STING pathway activation in tumor biopsies, immune cell infiltration (CD3^+^ T cells, macrophages), cytokine levels in CSF/serum, safety, and neurotoxicity	(Berger et al. (2022))
4	Intravenous TLR7/8‐agonist nanoparticles (myeloid‐targeting)	Mouse (immunocompetent)	Syngeneic GL261 orthotopic glioma	Intravenous β‐cyclodextrin nanoparticles loaded with R848 (CDNP‐R848), a dual vector, could mimic this systemic paradigm	MRI‐based tumor burden, survival, regression versus empty NP	Reprogramming of tumor‐associated macrophages/microglia: MHC‐II, CD86, M1/M2 markers, myeloid transcriptomics; demonstration of T cell‐independent tumor control	(Turco et al. (2023))
5	Local STING‐activating HA polymer in aggressive GBM	Mouse (immunocompetent)	SB28 orthotopic GBM (poorly immunogenic model)	Stereotactic intratumoral injection of hyaluronic acid–MSA2 STING polymer conjugate	Survival, tumor volume by MRI/bioluminescence, response versus free agonist	Intratumoral DC activation, CD8^+^ T cells, NK cells, Treg depletion, STING target engagement (p‐TBK1, IFN‐β) in situ	(Pesce et al. (2025))
6	3D GBM spheroids/organoids with microglia or myeloid cells	Human in vitro/ex vivo	GBM spheroids or patient‐derived organoids with added microglia, macrophages, or peripheral myeloid cells	Lipid or polymeric NPs carrying STING agonists ± microglia‐polarizing cargo (M1 skewing)	Spheroid size, viability, invasion into matrix, resistance to TMZ or RT	Microglial M1/M2 markers (CD86, iNOS, CD206, Arg1), cytokine and chemokine secretion, STING activation (p‐TBK1, IFN‐β) in myeloid compartment	(Hong et al. (2021))
7	3D microfluidic GBM‐microglia co‐culture	Human cells in vitro	U373MG + human microglia in a 3D microfluidic chip, plus 5 patient‐derived GBM lines	EVs loaded with miR‐124 could be replaced conceptually by your dual lipid nanovector	GBM proliferation, migration, invasion, TMZ chemosensitivity	M1/M2 microglia markers, STAT3 signaling, cytokine profile, and NK cell recruitment into the microfluidic chamber	(Hong et al. (2021))
8	Orthotopic GBM xenograft + exosomes	Mouse (immunodeficient)	Patient‐derived GBM or U87MG intracranial xenografts in nude mice	Systemic or local administration of DT‐Exo‐STING; adaptable to lipid nanovector via intravenous or intratumoral	Tumor burden (bioluminescence or MRI), overall survival, body weight	Infiltration of CD8^+^ T cells and NK cells, DC activation, STING activation in tumor and myeloid cells, cytokine panel (IFN‐β, CXCL10, CCL5)	(Bao (2024))
9	Patient‐derived GBM organoids + autologous immune cells	Human ex vivo	Patient‐derived GBM organoids co‐cultured with autologous PBMCs	Chimeric dendritic cell–tumor exosomes loaded with STING agonist (DT‐Exo‐STING)	Tumor cell killing in organoids, viability, IFN‐I–dependent growth control	STING pathway activation (p‐TBK1, p‐IRF3), type I IFN and ISG expression, CD8^+^ T‐cell activation, memory T‐cell markers	(Bao (2024))
10	Tumor‐targeted STING agonist ADC (non‐CNS)	Mouse (immunocompetent, various syngeneic tumors)	Several HER2^+^ and HER2^+^ low solid tumors (subcutaneous)	Intravenous STING agonist antibody–drug conjugates (STING‐ADC) versus free STING agonist	Tumor growth delay/regression, survival, synergy with anti‐PD‐L1	STING pathway activation in tumor cells and myeloid cells, type I and III IFN induction, M2 to M1 macrophage polarization, DC and T‐cell activation, serum cytokines (safety)	(Wu et al. (2022); Bukhalid et al. (2025))

### 8.3 Endpoints: Immunological, Oncogenic, and Safety Metrics

Barriers to glioblastoma immunotherapy are daunting and have slowed the pace of clinical and preclinical advancement. The immunosuppressive tumor microenvironment presents a particular obstacle, requiring delivery vehicles that cross the BBB and harbor functional agents to trigger STING activation and M1 polarization of Microglia to produce inflammatory cytokines (Bao 2024; Chen, Meng, et al. 2023).

## 9 Challenges, Risks, and Ethical Considerations

The approach is confronted with three interconnected challenges. First of all, there are safety questions: STING and its agonists should be shown not to interfere with the strong inflammatory sequelae observed in other systems (Wang, Liu, et al. 2022). Some of these influences might be harmful to nerve cells and homeostasis, leading to neurotoxicity (Chen, Xu, et al. 2024). Second, achieving off‐target delivery while maintaining CNS‐wide specificity is necessary to reduce peripheral immune activation and prevent side effects in other brain regions (Mallick et al. 2025). Thirdly, long‐term neurological sequelae need to be evaluated. Preclinical work must evaluate a wide range of side effects and neurologic safety, and determine the effects of dose escalation (Bartusik‐Aebisher et al. 2025).

Additionally, several manufacturing, scalability, reproducibility, regulatory, and translational challenges need to be addressed next. The current technologies for synthesizing the dual‐functional platform were ineffective and could hinder mass‐scale production (Raman et al. 2024; Buist et al. 2025). Complex compositions containing several biologically active agents, especially with different physico‐chemical properties and structures, make reproducibility even more challenging (Horta et al. 2025). The amount of work required to develop such formulas underscores the problem. Concomitantly, adherence to regulatory standards and awareness of approval trajectories would shape both technical development and timelines (Khot et al. 2024).

### 9.1 Safety of STING Agonism and Inflammation

The concerns associated with systemic cGAS‐STING agonism for glioblastoma therapy are twofold: safety of stimulation and neurotoxicity (Chen, Meng, et al. 2023). The two potential drawbacks of STING activation are proinflammatory immune responses and subsequent toxicities that can occur when initiating cGAS‐STING signaling, as well as overall safety concerns during delivery to the central nervous system (Low et al. 2024; Islam et al. 2024). Many reports indicate that cGAMP, the endogenous ligand of STING, is an important molecule for eliciting inflammatory responses and activating the cGAS‐STING pathway in model systems, and may also play a role in delivering cGAMP to the brain (Zhang, Li, et al. 2025). Administration of a STING agonist, for example, cyclic di‐AMP, to the glial compartment in animal models of glioblastoma has similarly been found to promote polarization of TAMs toward an M1 phenotype, thereby further increasing the immunogenicity of the TME (Zhang, Sun, et al. 2025).

### 9.2 Off‐Target Effects and Neurological Safety

Scientists reviewed and summarized chunked content on a double‐action lipid‐based nanovector technique against glioblastoma immunotherapy. Based on patterns found in references, the following section of the literature review is presented (Musielak and Krajka‐Kuźniak 2025). The lack of diverse mechanisms increases the risk of off‐target effects in bidirectional systems (Khot et al. 2024). First, regardless of STING activation, the initial cytosolic transfection of the chosen mRNA using the nanovector lipid is highly prone to nonspecific immune stimulation (Fu et al. 2025). Specifically, the non‐targeted mRNAs could be recognized in the cytosol by pattern recognition receptors (PRRs) like retinoic acid‐inducible gene I (RIG‐I) and TLR 3, whose activation will lead to the generation of type I interferon response (IFN‐mediated), as well as other pro‐inflammatory cytokines such as interleukin 6 (IL‐6) and TNF‐α, among others (Wang, Liu, et al. 2022). Second, substantial concerns remain regarding the long‐term tolerability of cGAS‐STING pathway activation in the CNS. Fortunately, nature rarely perpetuates inflammation to this degree throughout the life span. Gao et al. found that the local expression of inflammatory cytokines returned to basal levels in WT mice after a single exposure to DMXAA, a STING agonist (Turco et al. 2023). Third, even when targeting STING directly, there may be a theoretical risk of cross‐activation of other compartment‐targeted mRNAs if not properly engineered (Zhang 2023). If this were to happen, toxic payloads such as proto‐oncogene siRNAs would still induce neurotoxic side effects. In the CNS, these risks can be further reduced by applying a well‐engineered multi‐target delivery approach (Khot et al. 2024).

### 9.3 Manufacturing and Translational Barriers

The considerable challenges associated with the manufacture and translation of dual‐function lipid‐based nanovectors for glioblastoma immunotherapy broadly fall into four categories: the need for scalable production methods (Salvato and Marchini 2024), the ability to formulate systems that retain both physicochemical properties and biological functionality under storage and processing conditions (Horta et al. 2025), the ability to generate nanovectors that can be segregated from assembly intermediates and reactants before final formulation (Mandal et al. 2025), and an understanding of how regulatory agencies will classify the materials and processes involved within the context of existing guidelines (Conte et al. 2025). Statutory timelines for the development and approval of new assays, reagents, and materials are necessarily lengthy, and the possibility of unforeseen challenges in generating quality‐assured products further complicates the establishment of a plausible development schedule (Thomas and Rahman 2025). The fabrication of dual‐function systems is inherently more complex than creating nanovectors designed for only one paradigm. Moreover, the specificity of each design must be maintained during manufacture, enabling stringent control over physiological release rates, the spatial congruence of release sites with targeted cells, and the type of stimulus that actuates release (Wang, Liu, et al. 2022; Zhao 2024).

## 10 Outlook and Future Directions

Future advancements in glioblastoma immunotherapy will no doubt be based on progress toward simultaneous presentation of both T‐ and B‐cell antigens, as enabled by rationally engineered platforms that sequentially tackle one or more of the major biological bottlenecks contributing to this deadly disease. A practical blueprint for the next chapter of research should revolve around four interlocking goals (Eckert et al. 2025). First, antigen presentation has to be improved by potentiating DC maturation, augmenting tumor‐antigen cross‐presentation, and delivering tumoral antigens or neoantigen‐encoding nucleic acids into nanovector compositions (Pavani et al. 2026; Aikins et al. 2024). In this context, the activation of STING is particularly appealing because it not only directly amplifies type I interferon modulation of immune cells, such as DCs, but also modifies antigen‐antigen‐presenting cell interactions and increases the chances of productive CD^8+^ T‐cell priming (Ma 2021; Badani et al. 2025).

Second, future platforms should be engineered to improve effector T‐cell homing and retention within GBM lesions. This reprogramming of the tumor microenvironment to promote chemokine production, suppress local myeloid immunosuppression, and skew M2‐like microglia/macrophages towards an M1‐like inflammatory phenotype supporting T‐cell recruitment and function will be critical (Yi et al. 2025; Pavani et al. 2026; Mandan and Kanugo 2025). Third, penetration through the BBB and selective tumor accumulation still need to be improved for clinical translation. This can be accomplished by fine‐tuning particle size, surface charge, PEG‐lipid ratio, receptor‐targeting ligands (transferrin or angiopep‐derived motifs), and external‐triggered release methods, e.g., thermomagnetic activation (Horta et al. 2025; Sarmah et al. 2025; Shen 2024; Tang et al. 2024).

Fourth, one of the most promising directions for dual‐function lipid nanovectors is the co‐delivery of multiple drugs to achieve synergistic effects. Rather than delivering a STING agonist alone, future systems should be evaluated for co‐encapsulation of M1‐polarizing cues, neoantigen vaccines, checkpoint inhibitors, RNAi cargos, or immunogenic cell death‐inducing agents in order to generate coordinated innate and adaptive antitumor responses (Peng et al. 2025; Sun, Fu, et al. 2025; Xiong et al. 2025). Importantly, these next‐generation platforms should be tested in orthotopic, immunocompetent, and humanized GBM models using integrated endpoints that capture BBB transport, STING pathway activation, microglial repolarization, antigen presentation, T‐cell infiltration, survival benefit, and neurological safety (Thomas and Rahman 2025; Zannikou et al. 2025; Li, Xu, et al. 2026). These priorities, taken together, create a translational roadmap for developing lipid nanovectors that not only deliver cargo across the BBB but also reprogram the GBM microenvironment toward durable antitumor immunity. In this context, we anticipate that the most advanced future platforms will be characterized by those that can target immune activation, myeloid reprogramming, BBB transport, and regulated combinatorial delivery integrated within a single clinically translatable nanovector architecture.

## 11 Conclusion

In conclusion, this review emphasizes the potential dual function of lipid‐based nanovectors as an efficient targeted delivery system for therapeutics, capable of enacting a two‐pronged approach: activation of the cGAS–STING axis to promote innate and adaptive anti‐tumor immunity, and re‐education of TME‐localized microglia toward a pro‐inflammatory M1‐like phenotype to overcome local immunosuppression. The main contribution of this article is not simply to recount these approaches independently but to unify them into a coherent design concept for next‐generation GBM immunotherapy. In particular, this review makes three key contributions. It first provides a biological justification for combining STING activation with M1 microglial polarization in the GBM‐M. Second, it consolidates the essential engineering principles required to realize simultaneous cargo delivery and diagnostic agent within a single lipid nanovector, particularly how to develop cargo selection, lipid composition, BBB‐oriented targeting, and stimuli‐responsive release in parallel. Third, it reviews preclinical evaluation requirements, safety issues, and translational hurdles that need to be overcome before such systems can advance toward clinical use. All together, this review highlights a design‐centric conceptual roadmap toward the development of multifunctional nanovectors that can jointly circumvent both immune suppression and delivery barriers facing GBM. We expect that this holistic outlook will facilitate the logical design of more effective, clinically translatable nanoinmunotherapeutic approaches for glioblastoma.

## Author Contributions

All authors contributed to this work. Mohamed S. Nafie, Mohamed Khaled Diab, and Sherif Ashraf Fahmy conceived of this work. Mohamed S. Nafie, Mohamed Khaled Diab, and Sherif Ashraf Fahmy wrote the original draft of the article. Authors Mohamed S. Nafie, Mohamed Khaled Diab, and Sherif Ashraf Fahmy revised and commented on all versions of the article. Authors have approved the final version of the article. They warrant that the article is the authors' original work, hasn't received prior publication, and isn't under consideration for publication elsewhere.

## Consent

Authors have approved the article, including the authorship determination and order of authorship.

## Conflicts of Interest

The authors declare no conflicts of interest.

## Data Availability

The authors have nothing to report.
